# Hydrogen sulphide alleviates depression-like behaviors by suppressing the hippocampal necroptosis-neuroinflammation-KP imbalance axis

**DOI:** 10.3389/fphar.2025.1691204

**Published:** 2025-10-24

**Authors:** San-Qiao Yang, Yu-Hui Tang, Si-Min Chen, Fen Liu, Ping Zhang, Wei Zou, Bo Wang, Xiao-Qing Tang

**Affiliations:** ^1^ Department of Anesthesiology, The First Affiliated Hospital, Hengyang Medical School, University of South China, Hengyang, Hunan, China; ^2^ Institute of Neuroscience & Department of Physiology, Hengyang Medical School, University of South China, Hengyang, Hunan, China; ^3^ NHC Key Laboratory of Neurodegenerative Disease (University of South China), Hengyang, Hunan, China; ^4^ Department of Neurology, The Affiliated Nanhua Hospital, Hengyang Medical School, University of South China, Hengyang, Hunan, China; ^5^ The Second Affiliated Hospital, Brain Disease Research Institute of Neurological Medicine Center, Hengyang Medical School, University of South China, Hengyang, Hunan, China

**Keywords:** H_2_S, depression, necroptosis, neuroinflammation, kynurenine pathway

## Abstract

**Background:**

We have previously demonstrated that hydrogen sulfide (H_2_S) is sufficient to attenuate depressive-like behavior induced by chronic unpredictable mild stress (CUMS), but its underlying mechanisms remain largely unknown. It is well known that necroptosis is a trigger of inflammation and neuroinflammation-induced kynurenine pathway (KP) imbalance is the main pathogenesis of depression. Meanwhile, H_2_S plays a pivotal role in the inhibition of inflammatory pathways. Hence, the present study sought to investigate whether the antidepressant effect of H_2_S is attributable to the inhibition of the axis of hippocampal necroptosis-neuroinflammation-KP imbalance.

**Methods:**

The depression model of Sprague-Dawley (SD) rats was established by CUMS for four consecutive weeks. The expressions of necroptosis-related protein and KP-related protein were evaluated by Western blotting (WB). The levels of inflammatory factors were measured by enzyme-linked immunosorbent assay (ELISA). The hippocampal metabolites of tryptophan were determined by LC-MS/MS.

**Results:**

In this study, we found that H_2_S was capable of inhibiting necroptosis and neuroinflammation and correcting KP imbalance in the hippocampus of CUMS-exposed rats. The enhancement of necroptosis via overexpressing RIPK3 reversed the antagonistic role of H_2_S in the depressive-like behaviors of CUMS-exposed rats and the inhibitory effects of H_2_S on necroptosis, neuroinflammation, and KP imbalance in the hippocampus of CUMS-exposed rats. Furthermore, overexpressing indoleamine 2,3-dioxygenase 1 (IDO1) abolished the correcting effect of H_2_S on hippocampal KP imbalance and the attenuating effect of H_2_S on depressive-like behaviors in the CUMS-exposed rats.

**Conclusion:**

The antidepressant-like role of H_2_S is achieved by negatively regulating the axis of necroptosis-neuroinflammation-KP imbalance in the hippocampus.

## 1 Introduction

Depression is the most common psychiatric disease, with more than 300 million people suffering from depression worldwide, and the number of patients with depression is increasing (Davis et al., 2021). However, the therapeutic strategy for depression is unsatisfactory. Therefore, it is of great significance to explore new antidepressants. Interestingly, we have found that Hydrogen sulfide (H_2_S), as a novel gaseous signaling molecule, attenuates depressive-like behaviors caused by β2-microglobulin, sleep deprivation (Kang et al., 2021; S.-Q. Yang et al., 2022) and has an antidepressant effect on diabetes-related depression (Jiang et al., 2021), suggesting that H_2_S is a promising therapeutic approach for depression. Studies have shown that the antidepressant effects of H_2_S are closely associated with the modulation of the PI3K/AKT/mTOR, Sirt1, and cGAS-STING pathways (Pinna et al., 2025). However, the underlying mechanisms are not yet fully understood.

The kynurenine pathway (KP) is the major pathway of tryptophan metabolism (Cervenka et al., 2017; Xue et al., 2023). Accumulating evidence suggests that KP imbalance, which is characterized by an abnormal ratio of neurotoxic to neuroprotective metabolites in kynurenines, has been associated with the development of depression (Brown et al., 2021; Deng et al., 2021; Ou et al., 2023). It is worth noting that neuroinflammation, which is one of the major pathological hallmarks of depression, is a significant contributor to KP imbalance (Kim and Jeon, 2018; Mithaiwala et al., 2021; Réus et al., 2023; H. Yao et al., 2023). Meanwhile, clinical studies have shown that elevated levels of neuroinflammation or inflammatory cytokines are observed in patients with depression (Miller et al., 2009; Setiawan et al., 2015) and that anti-inflammatory drugs exert a certain therapeutic effect on depressive symptoms (Bauer et al., 2018; Tolkien et al., 2019). These findings suggest that the correction of KP imbalance via attenuating neuroinflammation is an effective approach to the prevention and treatment of depression. Importantly, evidence has emerged that H_2_S plays a pivotal role in inhibiting neuroinflammation (L. Cao et al., 2018; H. Zhao et al., 2017). It is therefore plausible to assume that H_2_S ameliorates depressive-like behaviors via suppressing neuroinflammation to correct KP imbalance.

What pathway is involved in the attenuating effect of H_2_S on neuroinflammation? It has been proposed that necroptosis promotes neuroinflammation in the pathogenesis of several neurodegenerative diseases (Hu et al., 2019; Picon et al., 2021; Yuan et al., 2019). While inhibition of necroptosis is capable of alleviating depressive-like behaviors (Sun et al., 2022). Therefore, necroptosis-mediated neuroinflammation plays a pivotal role in the pathogenesis of depression. Moreover, it has been suggested that cystine deprivation triggers necroptosis (Tang et al., 2016). As cystine is an essential substrate of endogenous H_2_S, we suggest that H_2_S can inhibit necroptosis. Therefore, it is feasible to speculate that inhibiting necroptosis contributes to the inhibitory role of H_2_S in the hippocampal neuroinflammation of CUMS-exposed rats.

In this study, we found that H_2_S inhibited necroptosis, alleviated neuroinflammation, and corrected KP imbalance in the hippocampus of CUMS-exposed rats. Enhancing hippocampal necroptosis via overexpressing RIPK3 abolished the antidepressant effect of H_2_S on depressive-like behaviors of CUMS-exposed rats and the correcting effect of H_2_S on KP imbalance in the hippocampus of CUMS-exposed rats. Furthermore, overexpressing IDO1 abolished the antidepressant-like effect of H_2_S on CUMS-exposed rats. Our present data demonstrate that the antidepressant-like effect of H_2_S is mediated by suppressing the axis of hippocampal necroptosis-neuroinflammation-KP imbalance.

## 2 Materials and methods

### 2.1 Animals

Adult male Sprague-Dawley rats (280–300 g), were purchased from the SJA Lab Animal Center of Changsha (Changsha, China). After adaptation for 1 week, rats were housed individually (21 °C ± 2 °C, relative humidity 50% ± 10%, 12-h light/12-h dark cycle), with access to water and food *ad libitum*. All the animal experiments were conducted following the National Institute of Health Guide for the Care and Use of Laboratory Animals (NIH Publications No. 80-23) revised in 1996 and approved by the Animal Use and Protection Committee of University of South China. To minimize the number of animals used and their suffering, rats were used according to the “3Rs” principles (Replacement, Reduction, and Refinement) in all experimental procedures.

### 2.2 Reagents

Sodium hydrosulfide (NaHS, a donor of H_2_S) was purchased from Sigma (Sigma, United States). BCA Protein Assay Kits (cat #P0009-1, Beyotime), RIPA lysis buffer (CWBIO, CW2334). RIPK3 Antibody (AF7942), Phospho-RIP3 Antibody (AF7443), MLKL Antibody (DF7412), and Phospho-MLKL Antibody (AF7420) were obtained from Affinity. RIPK1 Antibody (3493S), and Phospho-RIPK1 Antibody (53286S) were purchased from Cell Signaling Technology. KMO Antibody (ab233529), 3HAO Antibody (ab106436), and TNF alpha ELISA kit (ab100785) were purchased from Abcam. ACMSD Antibody (A15953), and QPRT Antibody (A14349) were purchased from Abclonal. IL-1 beta ELISA kit (RLB00) and IL-6 ELISA kit (R6000B) were purchased from R&D Systems. IL-4 ELISA kit (CSB-E04635r) and IL-10 ELISA kit (CSB-04595r) were purchased from CUSABIO. IDO1 Antibody (sc-137012) was purchased from Santa Cruz.

### 2.3 Drug administration

A total of 1.68 mg or 5.6 mg of NaHS was dissolved in 1 mL of phosphate-buffered saline (PBS) to equal concentrations of NaHS 30 or 100 μmol/mL, respectively. After successfully establishing the depression model, NaHS (30 μmol/kg or 100 μmol/kg) was injected intraperitoneally for 2 weeks. Following the administration, a sequence of behavioral experiments was carried out. The GSK872 was first dissolved in DMSO to prepare a stock solution, which was then diluted with artificial cerebrospinal fluid to achieve the final working concentration of 25 mM. The final concentration of DMSO in all treatments was less than 0.1%. A total of 1 μL of GSK872 was injected into the lateral ventricles (coordinates relative to the bregma: 1 mm posterior, 2 mm lateral, 4 mm deep) of the rats for 2 weeks to inhibit necroptosis.

### 2.4 Chronic unpredictable mild stress (CUMS) procedure

Rats exposed to CUMS were subjected to two types of mild stressors each day that were unpredictable in time and manner for 2 weeks. Mild stressors were as follows: (1) Food deprivation 24 h, (2) Water deprivation 24 h, (3) Cage tilt 7 h (45°), (4) Overnight illumination, (5) Habitation in a soiled cage 24 h (200 mL of water in 100 g of sawdust bedding), (6) Physical restraint 2 h, (7) Ice water swimming 5 min, (8) Tail suspension 5 min.

### 2.5 Adeno-associated virus (AAV) injection

SD rats were anesthetized by sodium pentobarbital (45 mg/kg, i.p.) and secured in a stereotaxic apparatus (Model 68302, RWD company, China). Using a 5-μL Hamilton syringe, 2 μL of AAV9-vector or AAV9-RIPK3 or AAV9-IDO1 suspension were slowly injected into the hippocampus over 5 min according to the following coordinates: (anteroposterior: –3.6 mm; left: +2.0 mm from bregma; depth: –4.0 mm from skin). To ensure the viral suspension can be injected completely into the hippocampus, the needle is left in place for two additional min before it is slowly removed. AAV9-vector or AAV9-RIPK3 or AAV9-IDO1 were stereotaxically injected into the hippocampus 2 weeks before the start of CUMS to allow for full transgene expression.

### 2.6 Behavioral tests

#### 2.6.1 Open field test (OFT)

The open field test (OFT) was performed to analyze spontaneous activity levels. Rats were placed into an arena (100 cm length × 100 cm width × 40 cm height) with black bottoms and walls. The total distances were automatically recorded for 5 min using a video-tracking system (JLBehv-LAR-1; Shanghai Jiliang Software Technology Co. Ltd.). The apparatus was cleaned with 75% ethanol before the test of the next subject.

#### 2.6.2 Novelty-suppressed feeding test (NSFT)

Novelty-suppressed feeding test (NSFT) was performed to analyze depressive-like behavior. Rats underwent food deprivation for 24 h before the test performance. On day 2, rats were placed into the testing cage containing the weighed food pellet in the center, and the time of first eating the food pellet was video recorded. To eliminate the differences in latency impacted by appetite-direct effects, the rat was reintroduced to their home cage, and the total food eaten during 10 min was measured.

#### 2.6.3 Sucrose preference test (SPT)

A sucrose preference test (SPT) was performed to assess the depressive-like behavior of rats. During the adaptive training stage, rats were first habituated to two identical bottles of 1% sucrose solution for 24 h and one was changed to water following 24 h. During the testing phase, Rats underwent food deprivation for 24 h and followed 12-h exposure to two identical bottles filled with either sucrose solution or water. Sucrose preference was calculated using the following equation: sucrose preference = the consumption of sucrose solution/(sucrose consumption + water consumption).

#### 2.6.4 Forced swimming test (FST)

The Forced swimming test (FST) was performed to assess depressive-like behavior. Rats were placed in a plastic cylinder (height: 60 cm, diameter: 30 cm) filled with 40 cm of water (25 °C ± 1 °C). Before the testing, each rat was habituated to swimming in the water-filled plastic cylinder for 15 min. During the test phase, the time of climbing, swimming, or immobility was recorded by a camera for 5 min. Following the test, the rat was removed from the cylinder, dried with a cloth towel, and returned to their home cages.

### 2.7 Liquid chromatography-tandem mass spectrometry (LC-MS/MS) analysis

Hippocampus tissue samples (100 ± 5 mg) were homogenized in 0.5 mL prechilled MS buffer (methanol/acetonitrile, 1:1, v/v; Guanghua Sci-Tech Co., Ltd., Guangzhou, China) 2 μL prechilled ultrapure water, followed by vortexing and sonication (20 min) in an ice-water ultrasonic bath. After incubation for 1–2 h at 4 °C and centrifugation at 14,000 g for 20 min at 4 °C, the supernatant was collected and dried under vacuum. For mass spectrometry detection, the collected supernatants were re-dissolved with 100 μL of acetonitrile/water (1:1, v/v) and then centrifuged at 14,000 g for 15 min at 4 °C. Subsequently, the supernatants were collected and were separated on an Agilent 1,290 Infinity LC system (Agilent Technologies, Loveland, CO, United States). Metabolites were acquired and quantified on an AB SCIEX Qtrap^®^ 5500 mass spectrometer (AB SCIEX, Framingham, MA) in the negative ion mode. The peak area and the retention times were extracted from ion chromatograms using MultiQuant (Sciex, Foster City, CA, United States). Finally, metabolites (quinolinic acid (QUIN), kynurenine (KYN), melatonin, 3-indolepropionic acid (IPA), picolinic acid (PIC), and nicotinamide adenine dinucleotide (NAD+), 3-hydroxy-kynurenine (3-HK), 5-hydroxy-L-Tryptophan, kynurenate acid) were identified by comparing the retention time to a purchased standard.

### 2.8 Western blot analysis

Collection of hippocampal tissue supernatants treated with RIPA lysis buffer (150 mmol/L NaCl, 20 mmol/L Tris-HCl, pH 7.5, 1% Triton X-100, 1 mmol/L phenyl methyl sulfonyl fluoride, 1 mmol/L Na3VO4, leupeptin, and EDTA). The protein concentration of each sample was measured by using BCA Protein Assay Kits (cat #P0009-1, Beyotime). Equivalent amounts of samples (50 μg) were mixed with 5× loading buffer and heated at 100 °C for 5 min. Then electrophoresis was performed using sodium dodecyl sulfate-polyacrylamide gel electrophoresis (SDS-PAGE). Subsequently, the separated proteins were electrotransferred to polyvinylidene fluoride (PVDF) membranes (Merck Millipore, MA, United States). The PVDF membranes were blocked with 5% skim milk in TBST for 2 h at 37 °C and then were separately incubated with primary antibodies at 4 °C overnight. After incubation, the membranes were washed three times in TBST and were incubated with a secondary antibody (1:5000) conjugated with horseradish peroxidase for 2 h. Finally, the membranes were visualized by autoradiographic films (Tanon-5600, Shanghai, China). The integrated optical density of the band was calculated by ImageJ software.

### 2.9 Statistical analysis

Statistical analysis was performed by SPSS soft version 20.0 (Chicago, IL, United States). Statistical comparisons were analyzed by repeated measurements for evaluation of within-group differences and one-way analysis of variance (ANOVA), followed by *Post hoc* Tukey’s test for multiple comparisons. The data are expressed as the mean ± standard error of the mean (SEM), and a *P-*value <0.05 was considered statistically significant.

## 3 Results

### 3.1 H_2_S inhibited the hippocampal necroptosis of CUMS-exposed rats

**FIGURE 1:**
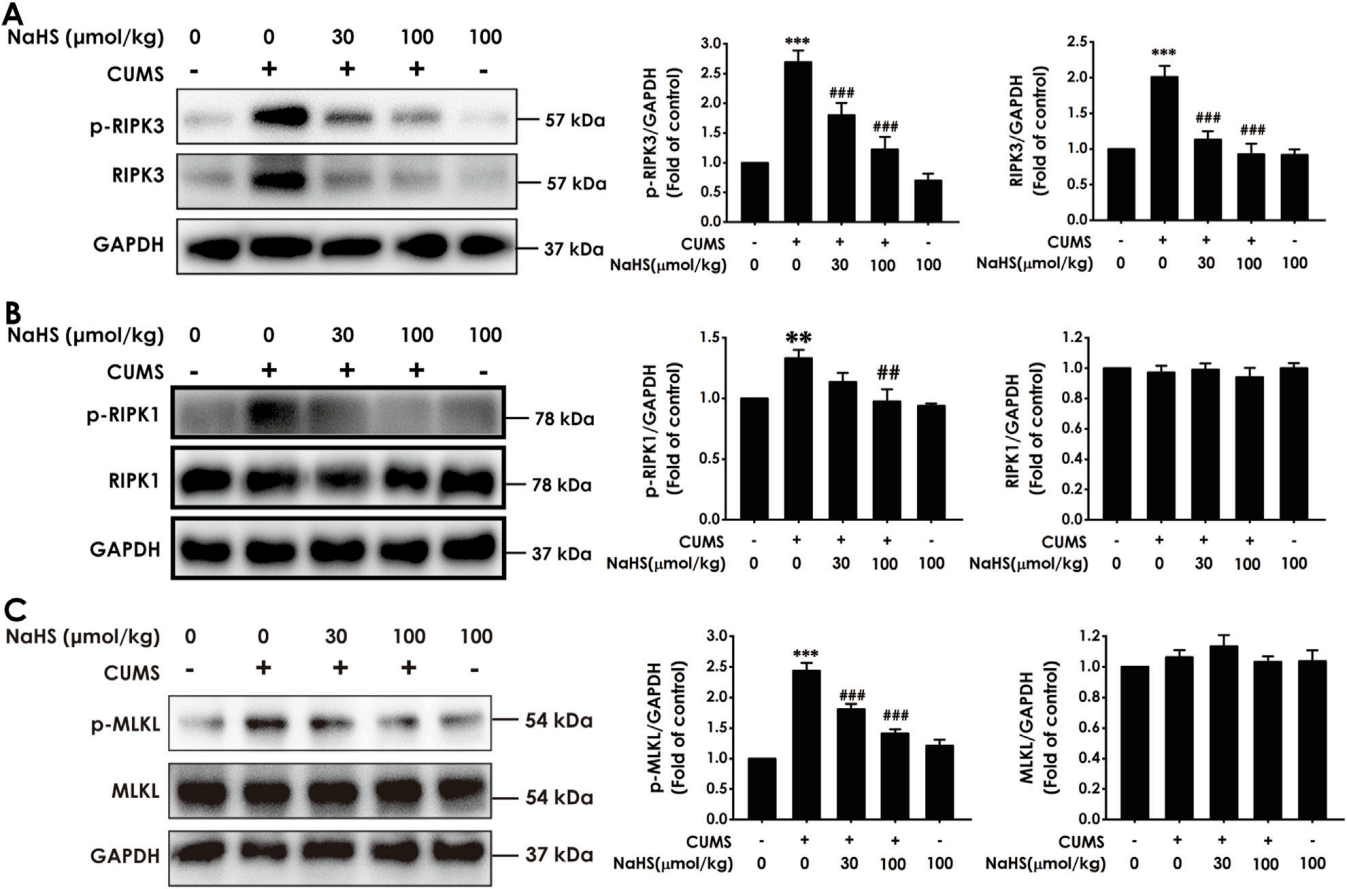
Effect of NaHS on the hippocampal necroptosis of CUMS-exposed rats. Rats were exposed to CUMS for 4 weeks and treated with NaHS (30, 100 μmol/kg, i.p.) for 2 weeks. The expressions of p-RIPK3, p-RIPK3 **(A)**, p-RIPK1, p-RIPK1 **(B)**, p-MLKL, and MLKL **(C)** in the hippocampus of rats were measured by Western blotting. Values are expressed as mean ± SEM (*n* = 3–5/group). ^**^ *P* < 0.01, ^***^ *P* < 0.001, versus the control group; ^##^ *P* < 0.01, ^###^ *P* < 0.001, versus the CUMS-exposed group.

To explore the antidepressant effect of H_2_S associated with the inhibition of necroptosis in the hippocampus of CUMS-exposed rats, we first observed the effect of H_2_S on the expression of necroptosis-related proteins in the hippocampus of CUMS-treated rats. The data show that CUMS-exposed rats exhibited elevated expressions of p-RIPK3 and RIPK3 (Figure 1A), p-RIPK1 (Figure 1B), and p-MLKL (Figure 1C) in the hippocampus, which were reversed by the exogenous administration of NaHS. However, there was no difference in the expression of RIPK1 and MLKL among the groups. Furthermore, we found that NaHS (100 μmol/kg) decreased the number of necrotic neurons in the hippocampus of CUMS-exposed rats (Supplementary Figure S1). Taken together, these findings indicate that H_2_S inhibits necroptosis in the hippocampus of CUMS-exposed rats.

### 3.2 H_2_S alleviated the hippocampal neuroinflammation of CUMS-exposed rats

**FIGURE 2:**
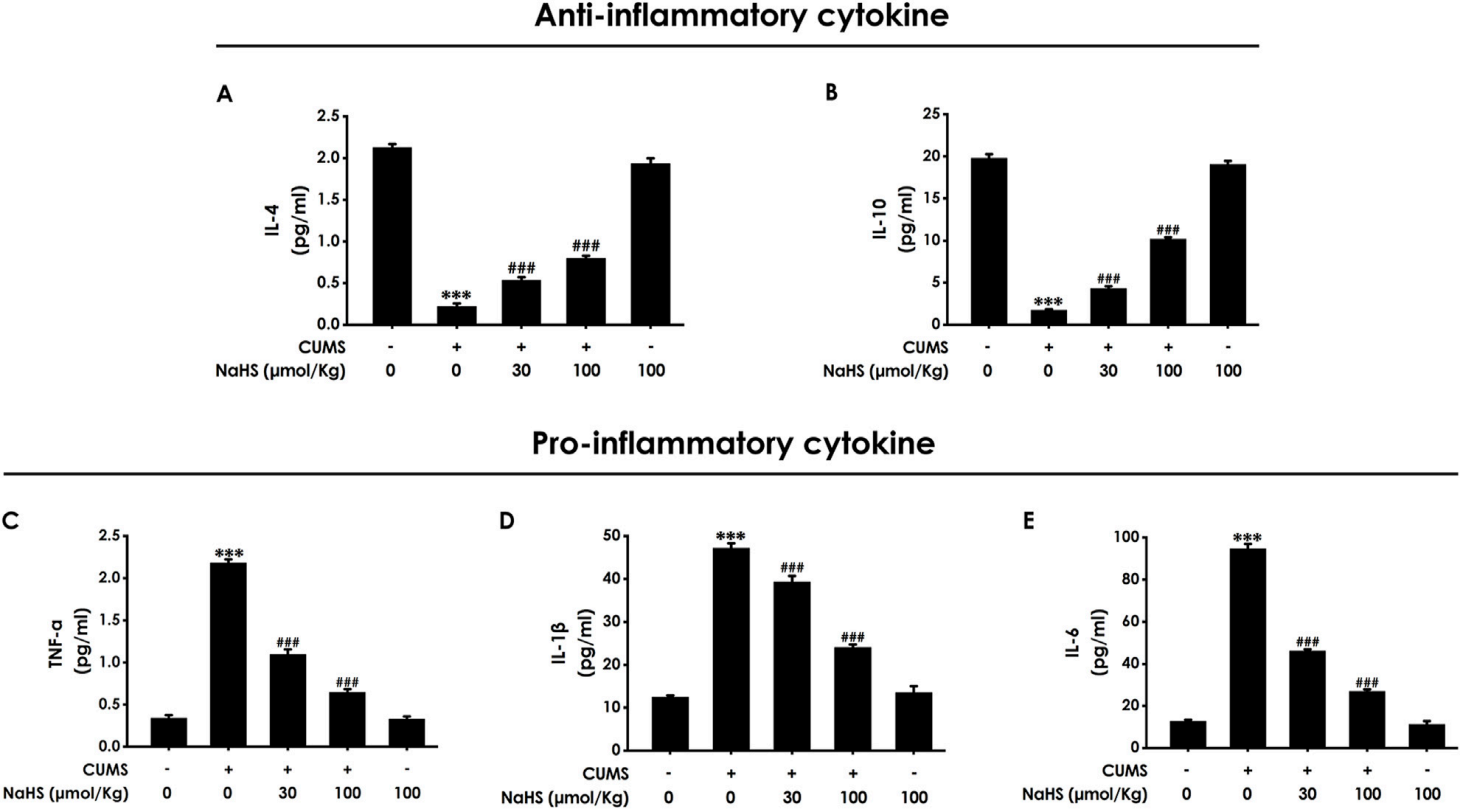
Effect of NaHS on the hippocampal neuroinflammation of CUMS-exposed rats. Rats were exposed to CUMS for 4 weeks and treated with NaHS (30, 100 μmol/kg, i.p.) for 2 weeks. The levels of IL-4 **(A)**, IL-10 **(B)**, TNF-α **(C)**, IL-1β **(D)**, and IL-6 **(E)** in the hippocampus of rats were detected by ELISA kits. Values are expressed as mean ± SEM (*n* = 3–5/group). ^***^ *P* < 0.001, versus the control group; ^###^ *P* < 0.001, versus the CUMS-exposed group.

It is well known that necroptosis is a pro-inflammatory form of cell death. Next, we will explore the effect of H_2_S on the neuroinflammation in the hippocampus of CUMS-exposed rats. ELISA results showed that NaHS treatment increased the levels of anti-inflammatory factors, including IL-4 (Figure 2A) and IL-10 (Figure 2B), while decreasing the levels of pro-inflammatory factors, including TNF-α (Figure 2C), IL-1β (Figure 2D), and IL-6 (Figure 2E) in the hippocampus of CUMS-exposed rats. These results suggest that H_2_S exerts an alleviating effect on neuroinflammation in the hippocampus of CUMS-exposed rats.

### 3.3 H_2_S corrected the imbalance of KP in the hippocampus of CUMS-exposed rats

**FIGURE 3:**
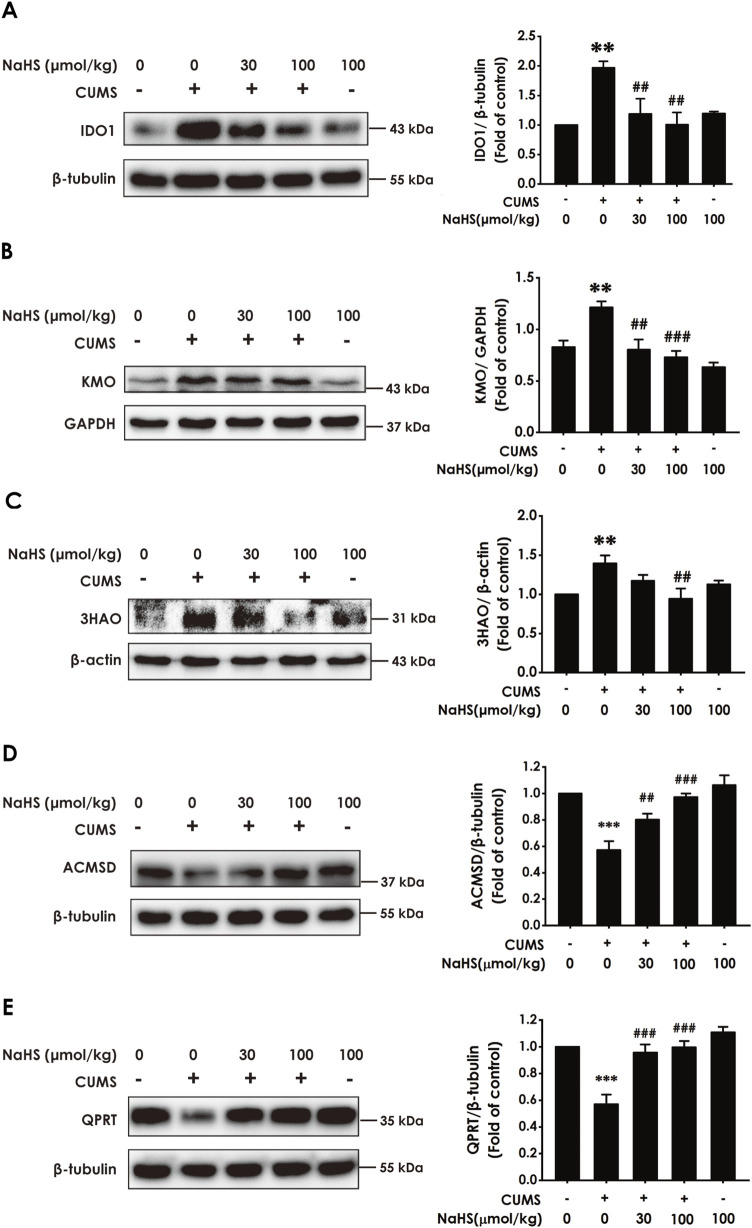
Effect of NaHS on the imbalance of KP in the hippocampus of CUMS-exposed rats. Rats were exposed to CUMS for 4 weeks and treated with NaHS (30, 100 μmol/kg, i.p.) for 2 weeks. The expression of IDO1 **(A)**, KMO **(B)**, 3HAO **(C)**, ACMSD **(D)**, and QPRT **(E)** in the hippocampus of rats was measured by Western blotting. Values are expressed as mean ± SEM (*n* = 3/group). ^**^ *P* < 0.01, ^***^ *P* < 0.001, versus the control group; ^##^ *P* < 0.01, ^###^ *P* < 0.001, versus the CUMS-treated group.

There is increasing evidence that pro-inflammatory cytokines drive the imbalance of KP and are closely associated with depression (Chen et al., 2023). Meanwhile, we have demonstrated that H_2_S has an alleviating effect on pro-inflammatory cytokine levels in the hippocampus of CUMS-exposed rats. Consequently, we further investigated the effect of H_2_S on the imbalance of KP in the hippocampus of CUMS-exposed rats. As expected, results showed that NaHS downregulated the expressions of IDO1 (Figure 3A), kynurenine 3-monooxygenase (KMO) (Figure 3B), and 3-hydroxyanthranilic acid 3,4-dioxygenase (3HAO) (Figure 3C), as well as upregulated the expressions of α-amino-β-carboxymuconate-ε-semialdehyde decarboxylase (AMCSD) (Figure 3D) and quinolinic acid phosphoribosyl transferase (QPRT) (Figure 3E) in the hippocampus of CUMS-exposed rats. In addition, LC-MS/MS results show that NaHS reduced the levels of quinolinic acid (QUIN) (Supplementary Figure S2A) and kynurenine (KYN) (Supplementary Figure S2B) and the ratio of KYN/TRP (Supplementary Figure S2C), as well as elevated the levels of melatonin (Supplementary Figure S2D), 3-indolepropionic acid (IPA) (Supplementary Figure S2E), picolinic acid (PIC) (Supplementary Figure S2F), and nicotinamide adenine dinucleotide (NAD+) (Supplementary Figure S2H) in the hippocampus of CUMS-exposed rats. Taken together, these data indicated that H_2_S corrected the imbalance of KP in the hippocampus of CUMS-exposed rats.

### 3.4 Overexpressing IDO1 in the hippocampus blocks H_2_S to correct the imbalance of KP in the hippocampus of CUMS-exposed rats

**FIGURE 4:**
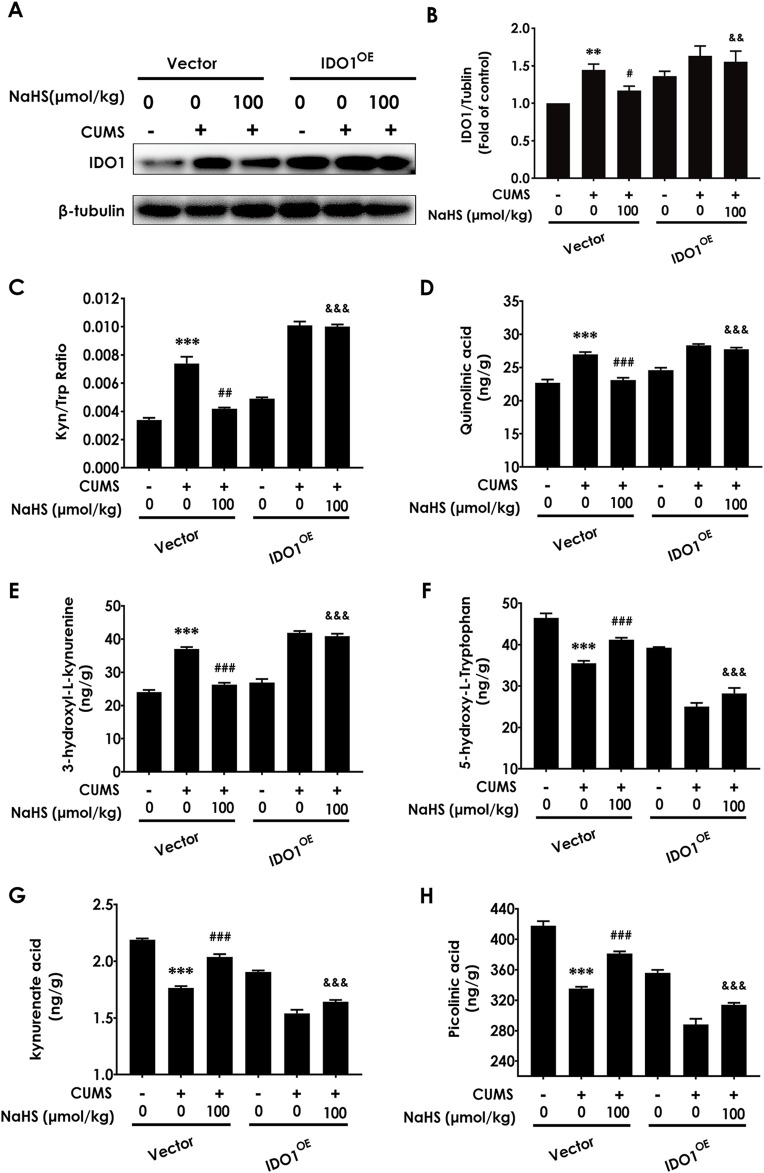
Effect of AAV-IDO1 on NaHS-correcting the imbalance of kynurenine metabolism in the hippocampus of CUMS-exposed rats. The expression of IDO1 **(A)** in the hippocampus of rats was measured by WB. The concentrations of KYN/TRP **(B)**, QUIN **(C)**, 3-HK **(D)**, 5-hydroxy-L-Tryptophan **(E)**, kynurenate acid **(F)**, and PIC **(G)** in the hippocampus of rats were measured by LC-MS/MS. The KYN/TRP **(H)** ratio represents IDO1 activity. Values are expressed as mean ± SEM (*n* = 3/group). **P < 0.01, ***P < 0.001 versus the vector group; ^#^ *P* < 0.05, ^##^ *P* < 0.01, ^###^ *P* < 0.001, versus co-treated with CUMS and vector group; ^&&^ *P* < 0.01, ^&&&^ *P* < 0.001, versus co-treated with CUMS, NaHS, and vector group.

To investigate the mediatory role of correcting the imbalance of KP in H_2_S-attenuated depressive-like behavior in CUMS-exposed rats, we should further detect whether abolishing H_2_S to correct the KP imbalance by overexpressing hippocampal IDO1 reverses the ameliorating role of H_2_S in the depressive-like behaviors of CUMS-exposed rats. Firstly, we confirm whether overexpressing IDO1 in the hippocampus abolishes H_2_S to correct the imbalance of KP in the hippocampus of CUMS-exposed rats. We found that the injection of AAV-IDO1 in the hippocampus upregulated the IDO1 expression (Figure 4A) and reversed the decreased effect of NaHS (100 μmol/kg) on the ratio of KYN/TRP (Figure 4B) and the levels of QUIN (Figure 4C) and 3-hydroxy-kynurenine (3-HK) (Figure 4D) and the increased effect of NaHS on the levels of 5-hydroxy-L-Tryptophan (Figure 4E), kynurenate acid (Figure 4F), and PIC(Figure 4G) in the hippocampus of CUMS-exposed rats. These results indicated that overexpressing IDO1 in the hippocampus abolished H_2_S to correct the imbalance of KP in the hippocampus of CUMS-exposed rats.

### 3.5 Overexpressing IDO1 in the hippocampus abolishes the inhibitory effects of H_2_S on the depressive-like behaviors of CUMS-exposed rats

**FIGURE 5:**
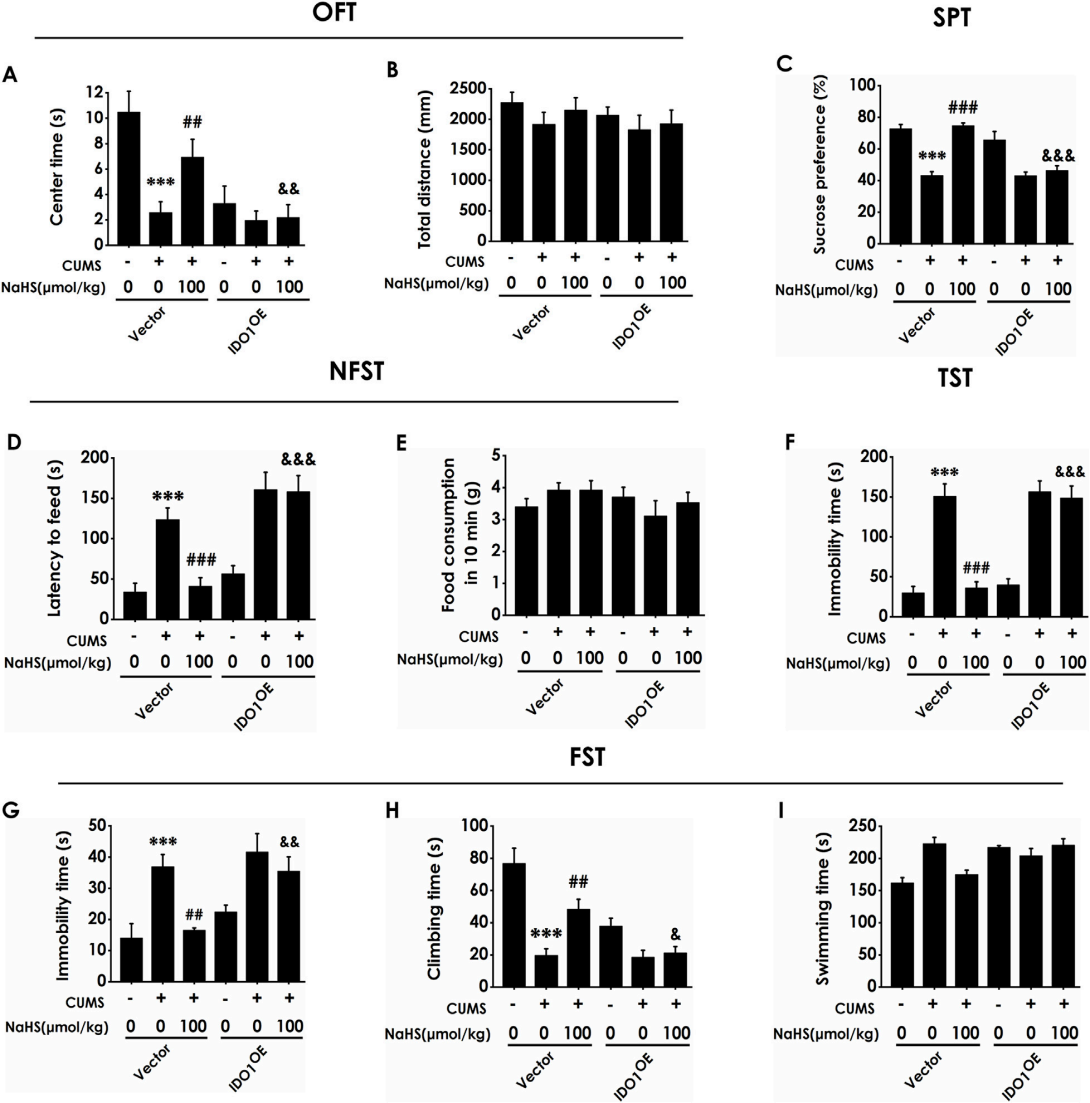
Effect of AAV-IDO1 on NaHS-prevented depressive-like behaviors of CUMS-exposed rats. Rats were injected with Vector or AAV-IDO1 into the hippocampus for once. One week after the injection of AAV-IDO1, rats were exposed to CUMS for 4 weeks and treated with NaHS (100 μmol/kg, i.p.) for 2 weeks. The depressive-like behaviors of rats were evaluated by OFT, SPT, NFST, TST, and FST. In the OFT, the center time **(A)** and the total distance **(B)** within 5 min were recorded. In the SPT, the sucrose preference **(C)** was recorded. In the NFST, the latency to feed **(D)** and food consumption in 10 min **(E)** were recorded. In the TST, the immobility time **(F)** within 5 min was recorded. In the FST, the immobility time **(G)**, climbing time **(H)**, and swimming time **(I)** within 6 min were recorded. Values are expressed as mean ± SEM (*n* = 8-15/group). ^***^ *P* < 0.001 versus the vector group; ^##^ *P* < 0.01, ^###^ *P* < 0.001, versus co-treated with CUMS and vector group; ^&^ *P* < 0.05, ^&&^ *P* < 0.01, ^&&&^ *P* < 0.001, versus co-treated with CUMS, NaHS, and vector group.

Next, we investigated whether overexpressing hippocampal IDO1 reverses the antidepressant-like role of H_2_S in CUMS-exposed rats. Injection of AAV-IDO1 in the hippocampus decreased the center time in the OFT in the rats cotreated with CUMS and NaHS (Figure 5A). However, three are no significant differences for total distance among groups in the OFT, which exclude the potential effect of motor ability on the experimental results (Figure 5B). In the SPT, injection of AAV-IDO1 in the hippocampus reduced the sucrose preference in the rats cotreated with CUMS and NaHS (Figure 5C). In the NSFT, injection of AAV-IDO1 in the hippocampus increased the latency to feed in the rats cotreated with CUMS and NaHS (Figure 5D), but did not influence the food consumption among groups (Figure 5E). In the TST, injection of AAV-IDO1 in the hippocampus prolonged the immobility time in the rats cotreated with CUMS and NaHS (Figure 5F). In the FST, we found that injection of AAV-IDO1 in the hippocampus increased the immobility time (Figure 5G) and decreased significantly the climbing time (Figure 5H) in the rats cotreated with CUMS and NaHS, but there is no difference for swimming time among groups (Figure 5I). Taken together, these data indicated that overexpressing IDO1 in the hippocampus abolished H_2_S to attenuate the depressive-like behaviors of CUMS-exposed rats.

### 3.6 Overexpressing RIPK3 in the hippocampus reversed the inhibitory effect of H_2_S on the hippocampal necroptosis of CUMS-exposed rats

**FIGURE 6:**
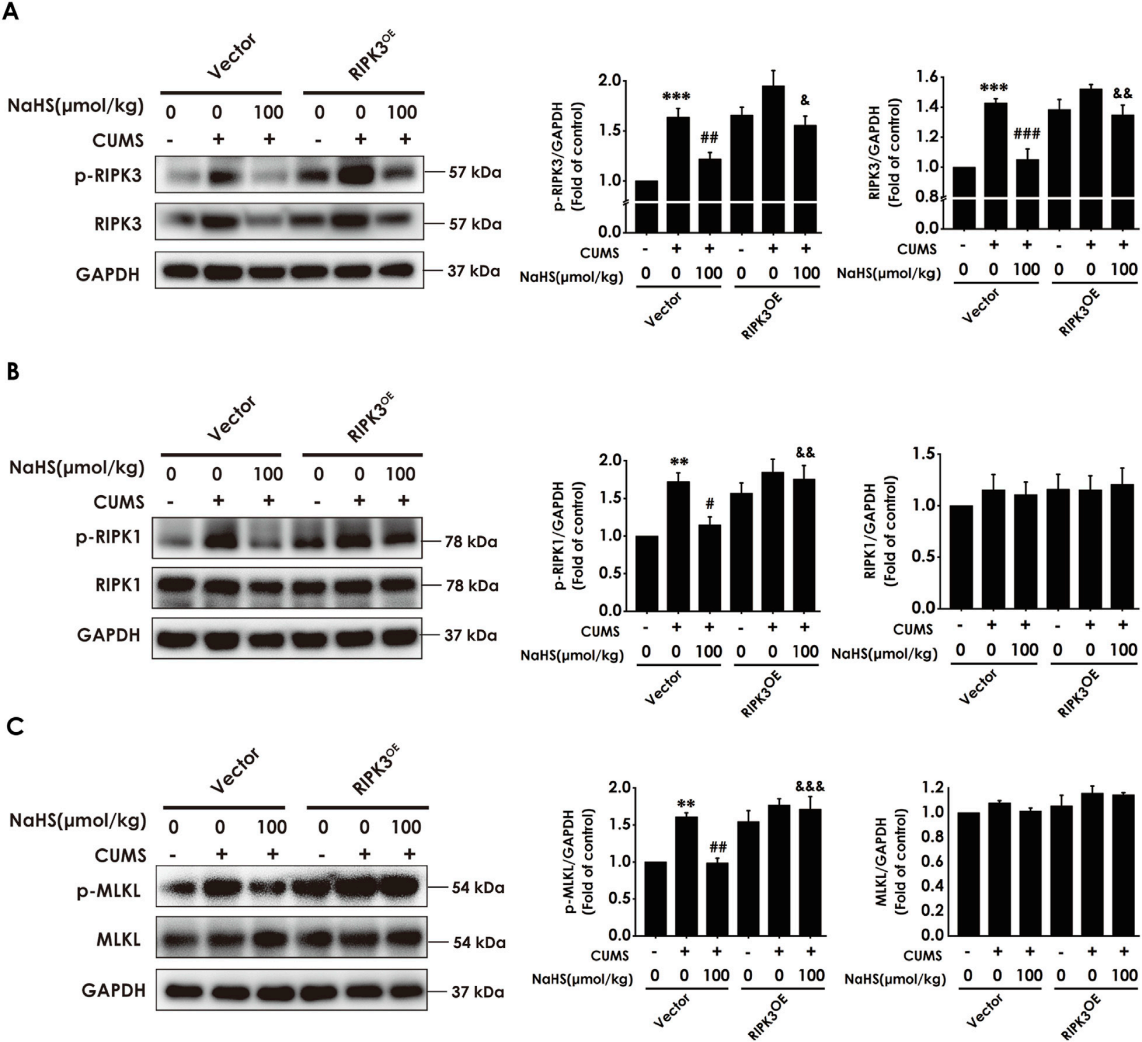
Effect of AAV-RIPK3 on NaHS-inhibiting necroptosis in the hippocampus of CUMS-exposed rats. Rats were injected with Vector or AAV-RIPK3 into the hippocampus once. One week after the injection of AAV-RIPK3, rats were exposed to CUMS for 4 weeks and treated with NaHS (100 μmol/kg, i.p.) for 2 weeks. The expressions of RIPK3 and p-RIPK3 **(A)**, RIPK1 and p-RIPK1 **(B)**, as well as MLKL and p-MLKL **(C)** in the hippocampus of rats were measured by WB. ^**^ *P* < 0.01, ^***^ *P* < 0.001 versus the vector group; ^#^ *P* < 0.05, ^##^ *P* < 0.01, versus co-treated with CUMS and vector group; ^&^ *P* < 0.05, ^&&^ *P* < 0.01, ^&&&^ *P* < 0.001, versus co-treated with CUMS, NaHS, and vector group.

To further define whether H_2_S is dependent on inhibiting hippocampal necroptosis to correct the imbalance of KP and ameliorate the depressive-like behavior, we will explore whether H_2_S-corrected the imbalance of KP and H_2_S-ameliorated depression are abolished by overexpressing hippocampal RIPK3 to reverse the inhibitory role of H_2_S on necroptosis in the hippocampus of CUMS-exposed rats. Firstly, we found that the injection of AAV-RIPK3 in the hippocampus abolished NaHS to decrease the number of necrotic neurons (Supplementary Figure S3) and to downregulate the expression of RIPK3, p-RIPK3 (Figure 6A), p-RIPK1 (Figure 6B), and p-MLKL (Figure 6C) in the hippocampus of CUMS-exposed rats, demonstrating that hippocampal RIPK3 overexpression reverses the inhibitory effect of H_2_S on necroptosis in the hippocampus of CUMS-exposed rats.

### 3.7 Overexpressing RIPK3 in the hippocampus reversed the anti-neuroinflammatory role of H_2_S in the hippocampus of CUMS-exposed rats

**FIGURE 7:**
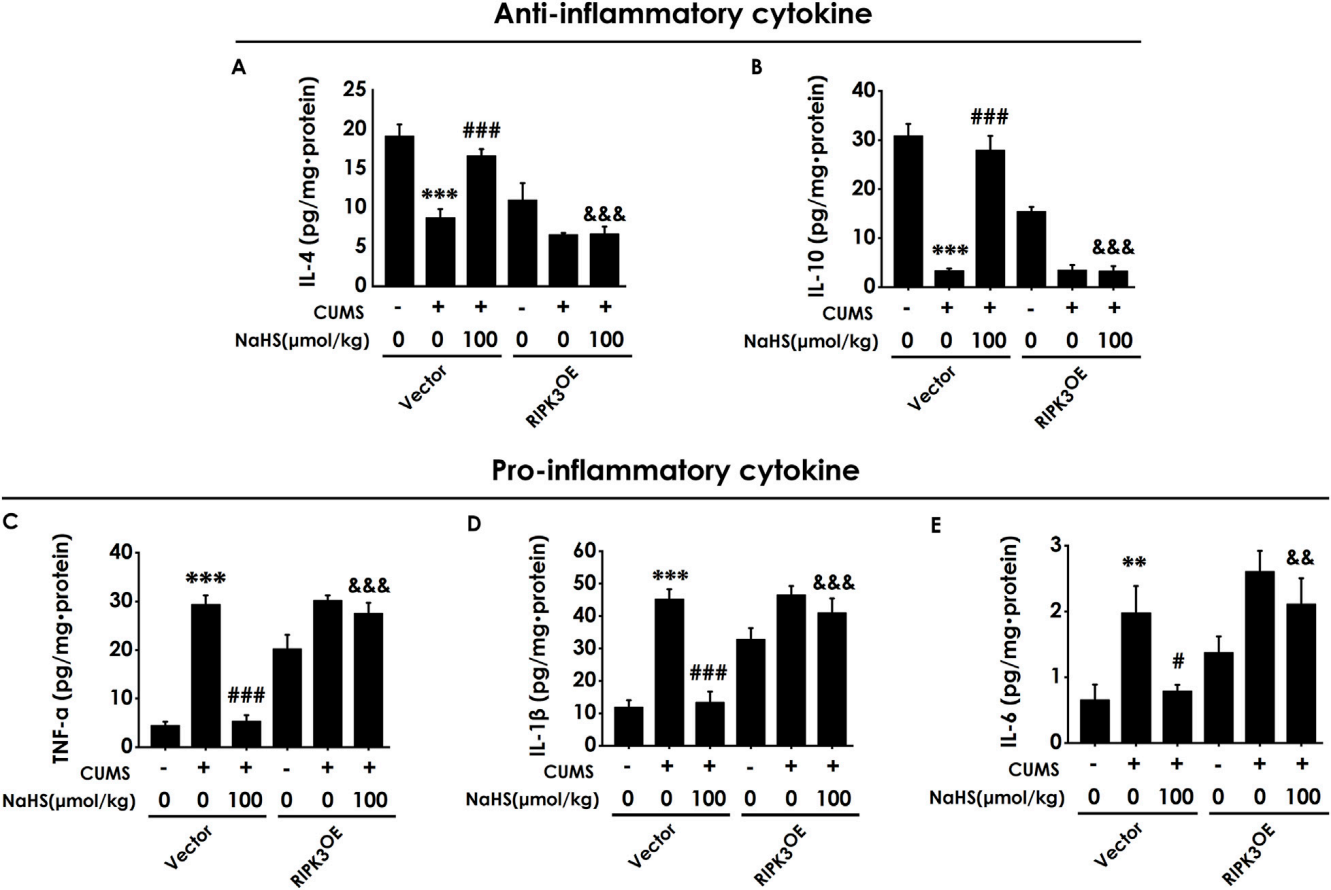
Effect of AAV-RIPK3 on NaHS-inhibited neuroinflammation in the hippocampus of CUMS-exposed rats. The levels of IL-4 **(A)**, IL-10 **(B)**, TNF-α **(C)**, IL-1β **(D)**, and IL-6 **(E)** in the hippocampus of rats were detected by ELISA kits.^**^ *P* < 0.01, ^***^ *P* < 0.001 versus the vector group; ^#^ *P* < 0.05, ^###^ *P* < 0.001, versus co-treated with CUMS and vector group; ^&^ *P* < 0.05, ^&&^ *P* < 0.01, ^&&&^ *P* < 0.001, versus co-treated with CUMS, NaHS, and vector group.

To investigate whether the anti-neuroinflammatory role of H_2_S in the hippocampus of CUMS-exposed rats relies on the inhibition of hippocampal necroptosis, we next explored whether the injection of AAV-RIPK3 in the hippocampus abolishes H_2_S to alleviate the hippocampal neuroinflammation of CUMS-exposed rats. As exhibited in Figure 7, overexpressing RIPK3 in the hippocampus blocked H_2_S to increase the levels of anti-inflammatory cytokines, IL-4 (Figure 7A) and IL-10 (Figure 7B), as well as to reduce the levels of pro-inflammatory cytokines, TNF-α (Figure 7C), IL-1β (Figure 7D), and IL-6 (Figure 7E), in the hippocampus of CUMS-exposed rats. Taken together, these data demonstrated that RIPK3 overexpression in the hippocampus reverses the alleviating effect of H_2_S on neuroinflammation in the hippocampus of CUMS-exposed rats.

### 3.8 Overexpressing RIPK3 in the hippocampus reversed H_2_S to correct the imbalance of KP in the hippocampus of CUMS-exposed rats

**FIGURE 8:**
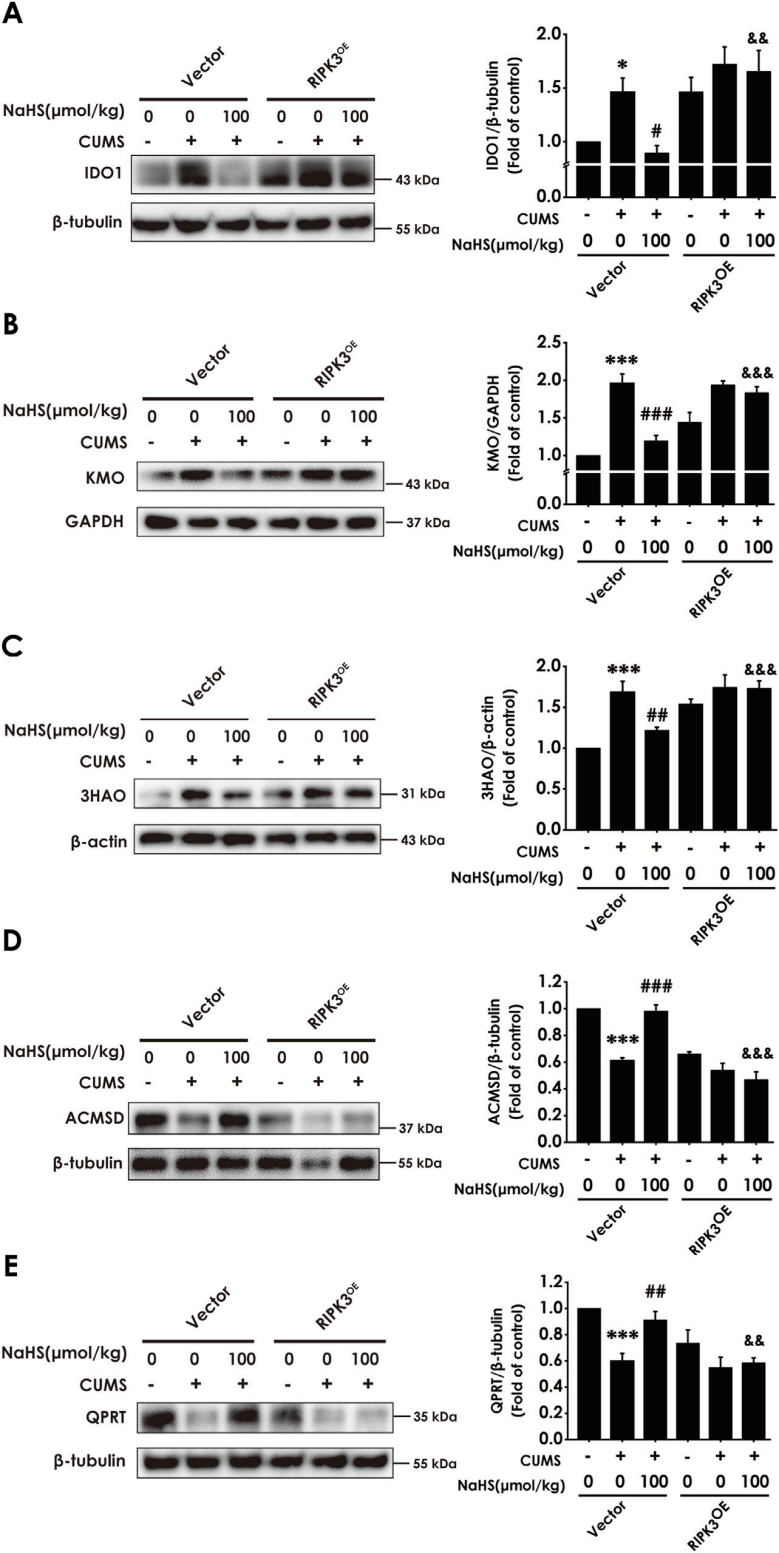
Effect of AAV-RIPK3 on NaHS-correcting the imbalance of KP in the hippocampus of CUMS-exposed rats. The expression of IDO1 **(A)**, KMO **(B)**, 3HAO **(C)**, ACMSD **(D)**, and QPRT **(E)** in the hippocampus of rats was measured by WB. Values are expressed as mean ± SEM (*n* = 3/group). ^*^ *P* < 0.05, ^***^ *P* < 0.001 versus the vector group; ^#^ *P* < 0.05, ^##^ *P* < 0.01, ^###^ *P* < 0.001, versus co-treated with CUMS and vector group; ^&&^ *P* < 0.01, ^&&&^ *P* < 0.001, versus co-treated with CUMS, NaHS, and vector group.

We further tested whether enhancing necroptosis via overexpressing hippocampal RIPK3 abolishes the role of H_2_S in correcting the hippocampal imbalance of KP in the CUMS-exposed rats. In this study, we found that the injection of AAV-RIPK3 in the hippocampus reversed H_2_S to downregulate the expressions of IDO1 (Figure 8A), KMO (Figure 8B), and 3HAO (Figure 8C), as well as to upregulate the expressions of ACMSD (Figure 8D) and QPRT (Figure 8E), in the hippocampus of CUMS-exposed rats. In addition, the injection of AAV-RIPK3 in the hippocampus blocked H_2_S to increase the levels of melatonin (Supplementary Figure S4A), serotonin (Supplementary Figure S4B), IPA (Supplementary Figure S4C), NAD+ (Supplementary Figure S4D), and PIC (Supplementary Figure S4E), as well as to decrease the level of N-formyl-kynurenine (Supplementary Figure S4F) and the ratio of KYN/TRP (Supplementary Figure S4G), in the hippocampus of CUMS-exposed rats. These results demonstrated that overexpressing hippocampal RIPK3 abolished H_2_S to correct the hippocampal imbalance of KP in the CUMS-exposed rats.

### 3.9 The overexpression of RIPK3 in the hippocampus abolishes the inhibitory effects of H_2_S on the depressive-like behaviors of CUMS-exposed rats

**FIGURE 9:**
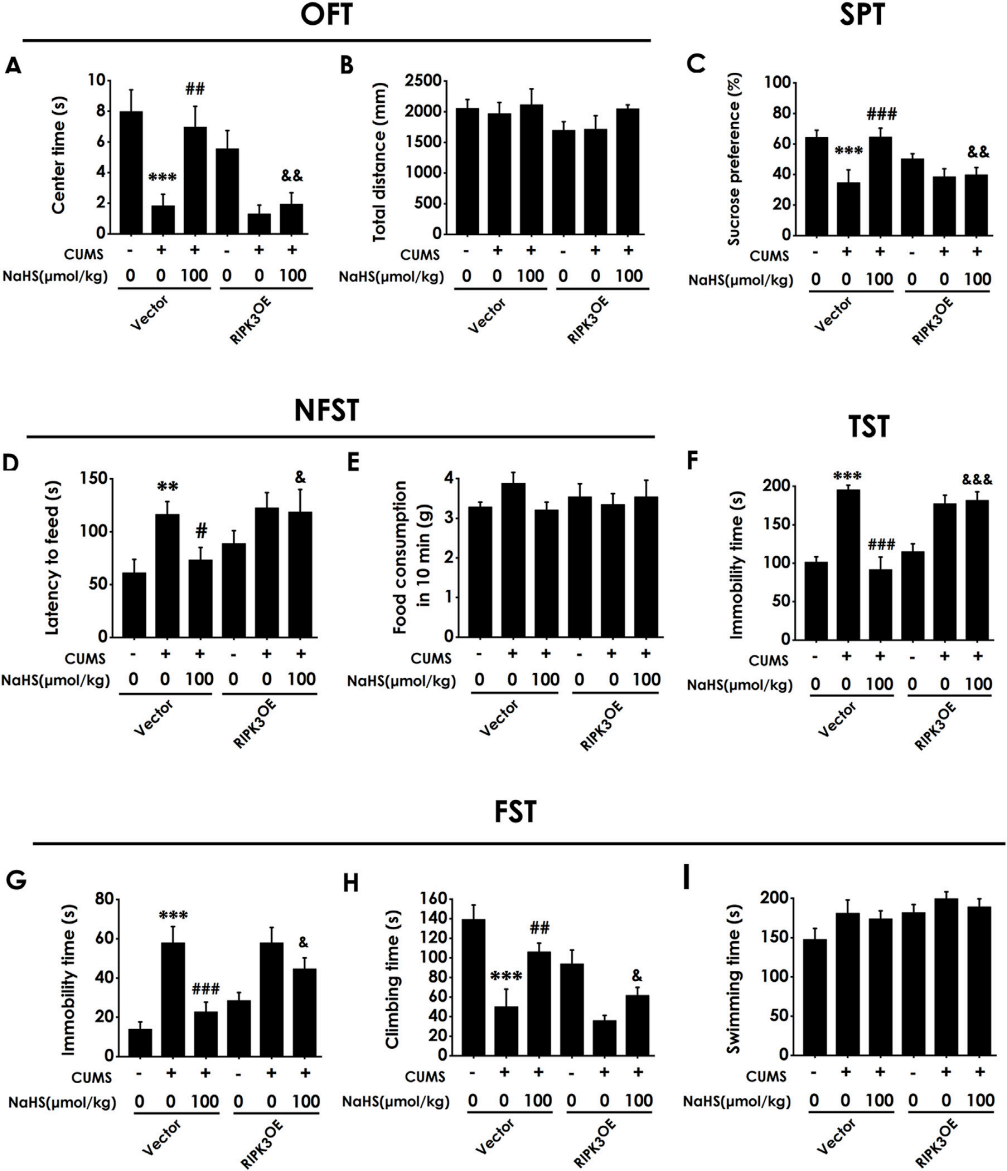
Effect of AAV-RIPK3 on NaHS-prevented CUMS-induced depressive-like behaviors of rats. The depressive-like behavior of rats was evaluated by OFT, SPT, NFST, TST, and FST. In the OFT, the center time **(A)** and total distance **(B)** within 5 min (min) were recorded. In the SPT, the sucrose preference **(C)** was recorded. In the NFST, the latency to feed **(D)** and food consumption in 10 min **(E)** were recorded. In the TST, the immobility time **(F)** within 5 min was recorded. In the FST, the immobility time **(G)**, climbing time **(H)**, and swimming time **(I)** within 6 min were recorded. Values are expressed as mean ± SEM (*n* = 8-15/group). ^**^ *P* < 0.01, ^***^ *P* < 0.001 versus the vector group; ^#^ *P* < 0.05, ^##^ *P* < 0.01, ^###^ *P* < 0.001, versus co-treated with CUMS and vector group; ^&^ *P* < 0.05, ^&&^ *P* < 0.01, ^&&&^ *P* < 0.001, versus co-treated with CUMS, NaHS, and vector group.

To confirm whether the antidepressant-like role of H_2_S is dependent on inhibiting hippocampal necroptosis, we detected the reversed role of the overexpression of hippocampal RIPK3 in H_2_S-inhibited depressive-like behaviors in the CUMS-exposed rats. Injection of AAV-RIPK3 in the hippocampus decreased the center time in the OFT in the rats cotreated with CUMS and NaHS (Figure 9A). However, there are no significant differences in total distance among groups in the OFT (Figure 9B), which excludes the potential effect of motor ability on the experimental results. In addition, injection of AAV-RIPK3 in the hippocampus reduced the sucrose preference in the SPT in the rats cotreated with CUMS and NaHS (Figure 9C). Injection of AAV-RIPK3 in the hippocampus prolonged the latency to feed in the NSFT in the rats cotreated with CUMS and NaHS (Figure 9D), but did not influence the food consumption among groups (Figure 9E). In the TST, injection of AAV-RIPK3 in the hippocampus increased the immobility time in the rats cotreated with CUMS and NaHS (Figure 9F). In the FST, we also found that injection of AAV-RIPK3 in the hippocampus increased the immobility time (Figure 9G) and decreased significantly the climbing time (Figure 9H) in the rats cotreated with CUMS and NaHS, but there is no difference for swimming time among groups (Figure 9I). Taken together, these data indicated that overexpressing hippocampal RIPK3 blocked H_2_S to ameliorate the depressive-like behaviors of CUMS-exposed rats.

### 3.10 Inhibiting hippocampal necroptosis ameliorates depressive-like behaviors in CUMS-exposed rats

**FIGURE 10:**
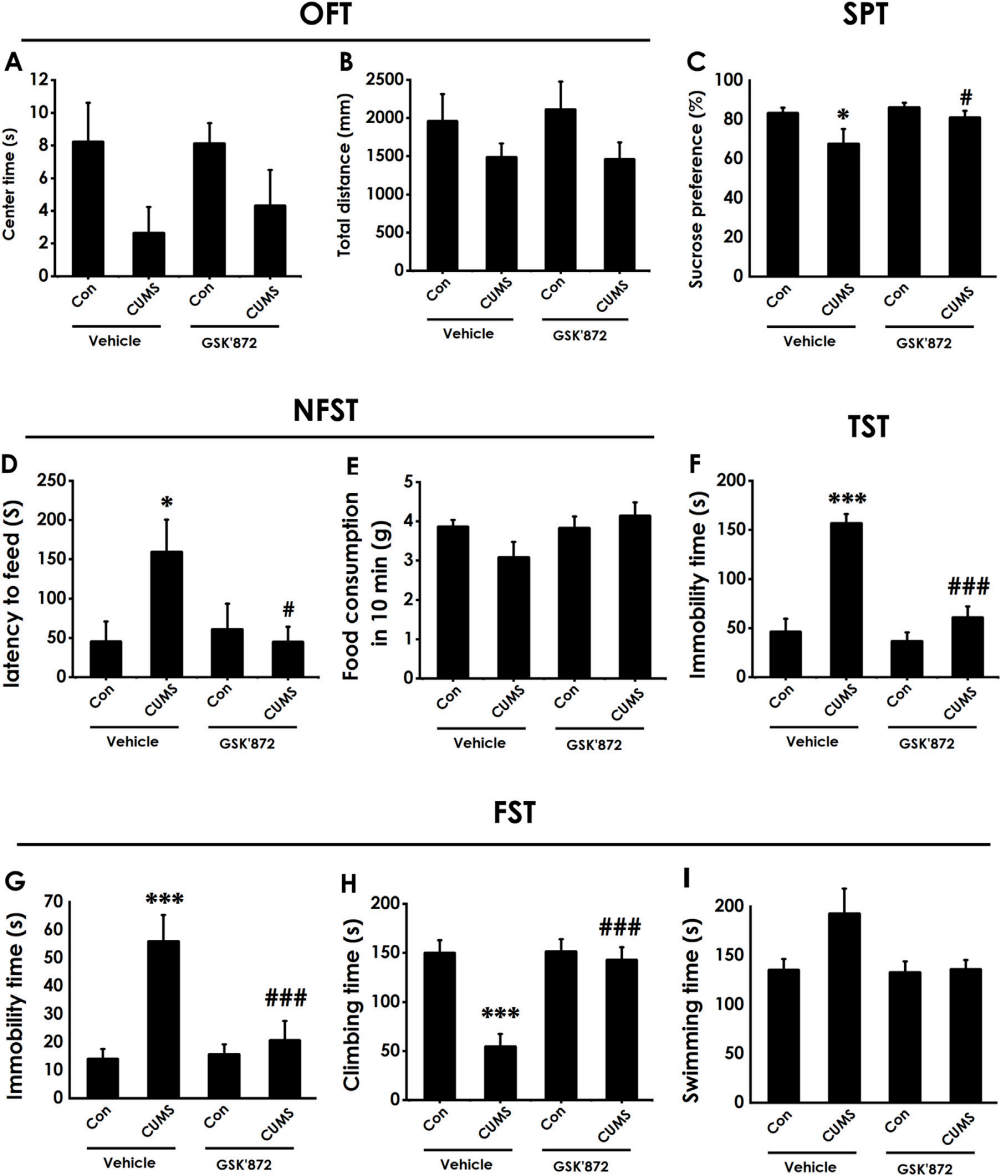
Effect of GSK872 on the depressive-like behaviors of CUMS-exposed rats. After 2 weeks of CUMS, rats were co-treated with GSK872 (25 mM, 1 ul, i.c.v) for 2 weeks. The depressive-like behaviors of rats were evaluated by OFT, SPT, NFST, TST, and FST. In the OFT, the center time **(A)** and total distance **(B)** within 5 (min) were recorded. In the SPT, the sucrose preference **(C)** was recorded. In the NFST, the latency to feed **(D)** and food consumption in 10 min **(E)** were recorded. In the TST, the immobility time **(F)** within 5 min was recorded. In the FST, the immobility time **(G)**, climbing time **(H)**, and swimming time **(I)** within 6 min were recorded. Values are expressed as mean ± SEM (*n* = 8–10/group). ^*^ *P* < 0.05, ^***^ *P* < 0.001, versus the control group; ^#^ *P* < 0.05, ^###^ *P* < 0.001, versus the CUMS treated group.

To further confirm whether inhibiting necroptosis is capable of ameliorating depression, we will explore whether GSK872, a RIPK3 inhibitor, ameliorates the depressive-like behavior of CUMS-exposed rats. Intracerebroventricular (i.c.v) injection of GSK872 increased the center time of CUMS-exposed rats (Figure 10A). However, there are no significant differences for total distance among groups in the OFT (Figure 10B), which excludes the potential effect of motor ability on the experimental results. In addition, GSK872 increased the sucrose preference in the SPT (Figure 10C) and reduced the latency to feed in the NSFT (Figure 10D) in the CUMS-exposed rats, but did not influence the food consumption among groups (Figure 10E). In the TST, injection of GSK872 decreased the immobility time of CUMS-exposed rats(Figure 10F). We also found that GSK872 reduced the immobility time (Figure 10G) and increased the climbing time (Figure 10H) in the CUMS-exposed rats in the FST. Still, there is no difference in swimming time among all groups (Figure 10I). Collectively, these results suggest that inhibiting necroptosis ameliorates the depressive-like behaviors in CUMS-exposed rats.

## 4 Discussion

We have previously demonstrated that H_2_S exerts an antidepressant-like effect on CUMS-exposed rats, but the underlying mechanisms are not yet fully understood. In this study, we aimed to explore whether suppressing the axis of necroptosis-neuroinflammation-KP imbalance in the hippocampus mediates the antidepressant-like role of H_2_S. We first demonstrated that H_2_S attenuated the levels of necroptosis and neuroinflammation and corrected the KP imbalance in the hippocampus of CUMS-exposed rats. Next, we revealed that the overexpression of IDO1 to abolish the correcting effect of H_2_S on the hippocampal KP imbalance reversed the attenuating effects of H_2_S on depressive-like behaviors in the CUMS-exposed rats. Furthermore, overexpression of RIPK3 abolished the inhibitory effect of H_2_S on hippocampal necroptosis. This reversal, in turn, blocked the beneficial effects of H_2_S, including the attenuation of depressive-like behaviors and neuroinflammation and the correction of KP imbalance in the hippocampus. These findings suggest that the antidepressant-like effects of H_2_S are dependent on suppressing the axis of hippocampal necroptosis-neuroinflammation-KP imbalance.

There is increasing evidence that neuroinflammation is closely associated with the onset of depression (Guo et al., 2023; Meyer et al., 2020; H. Yao et al., 2023). Preclinical and clinical studies have shown that the treatment of anti-inflammatory drugs positively attenuates depressive symptoms (Z.-Y. Cao et al., 2020; W. Li et al., 2021). It has been confirmed that H_2_S plays an important in the attenuation of neuroinflammation (T.-T. Li et al., 2022; X. Li et al., 2023; Y. Zhao et al., 2024), but the attenuating effect of H_2_S on neuroinflammation in the hippocampus of CUMS-exposed rats remains indefinite. Our study has shown that the treatment of NaHS reduced the levels of pro-inflammatory factors and increased the levels of anti-inflammatory factors in the hippocampus of CUMS-exposed rats, suggesting that the antidepressant role of H_2_S is associated with the attenuation of hippocampal neuroinflammation.

It was recently reported that necroptosis, programmed cell death, has been recognized as a pro-inflammatory form of cell death (Heckmann et al., 2019; Yu et al., 2021), which has been implicated in the pathogenesis of central nervous system disease (Ito et al., 2016; Thadathil et al., 2021; Yuan et al., 2019). It is worth noting that cystine deprivation, resulting in the decrease of H_2_S level, triggers necroptosis (Tang et al., 2016). Therefore, examining whether H2S attenuates neuroinflammation by inhibiting hippocampal necroptosis is interesting. Our study found that the treatment of NaHS inhibited the necroptosis in the hippocampus of CUMS-exposed rats. Of note, the treatment of NaHS only decreased the total protein levels of RIPK3 in the hippocampus of CUMS-exposed rats but did not influence the total protein levels of RIPK1 and MLKL in the hippocampus of CUMS-exposed rats. Furthermore, overexpressing hippocampal RIPK3 via injecting AAV-RIPK3 reversed the attenuated effect of NaHS on depressive-like behavior and the inhibited effect of H_2_S on necroptosis-mediated neuroinflammation, indicating that the inhibited effect of H_2_S on necroptosis is dependent on the decrease of RIPK3. Finally, we demonstrated that the inhibition of necroptosis by the injection of GSK872, a selective RIPK3 inhibitor, alleviated depressive-like behaviors of rats. Moreover, it has been previously reported that the inhibition of necroptosis alleviates depressive-like behaviors of mice (Sun et al., 2022), suggesting that inhibiting necroptosis is an important target for the treatment of depression. Further supporting our results, other studies have identified additional molecular mechanisms underlying the antidepressant effects of H_2_S in models of diabetes and inflammation, including the reduction of ferroptosis and neuroinflammation (Wang et al., 2021), and inhibition of the NF-κB/NLRP3 inflammasome and pyroptosis, along with the preservation of mitochondrial function in the hippocampus (Bao et al., 2023). Taken together, these results suggested that H_2_S is dependent on inhibiting hippocampal necroptosis to attenuate the hippocampal neuroinflammation and depressive-like behaviors in CUMS-exposed rats and identified RIPK3 as a potential therapeutic target for depression.

Emerging evidence suggests that inflammation triggered the KP imbalance via enhancing the expressions of KP-related rate-limiting enzymes (Mithaiwala et al., 2021; Schwieler et al., 2015; Zunszain et al., 2012). KP imbalance resulting from the overactivation of KP-related rate-limiting enzymes usually is accompanied by elevated levels of neurotoxic metabolites, quinolinic acid (QUIN), and diminished levels of neuroprotective metabolites, serotonin, melatonin, and picolinic acid (PA) (Fujigaki et al., 2017; Hestad et al., 2022; Paul et al., 2022; Yan et al., 2015). KP imbalance is widely acknowledged as an important pathological mechanism of depression (Deng et al., 2021; Joisten et al., 2021; Paul et al., 2022), indicating that the correction of KP imbalance is an effective approach to the prevention and treatment of depression. Given the inhibiting effect of H_2_S on necroptosis-mediated neuroinflammation and the activating effect of inflammation on KP imbalance, we investigated the effect of H_2_S on the KP imbalance in the hippocampus of CUMS-exposed rats. In this study, we found that H_2_S increased the level of melatonin, IPA, PA, and NAD+ and decreased the level of QUIN, KYN, and the ratio of KYN/TRP via downregulating the expressions of IDO1, KMO, and 3HAO and upregulating the expressions of AMCSD and QPRT in the hippocampus of CUMS-exposed rats. In addition, overexpressing IDO1 in the hippocampus reversed the attenuating effect of H_2_S on depressive-like behaviors and the correcting effect of H_2_S on KP imbalance in CUMS-exposed rats. Consistent with our finding, it has been confirmed that decreasing the level of QUIN (Schwieler et al., 2016) or increasing the level of melatonin, IPA, PA, and NAD+ contributes to attenuating depressive symptoms (Aarsland et al., 2019; Ali et al., 2020; D. Yao et al., 2023; Zhang et al., 2022). Our results, which show that H_2_S negatively regulates the kynurenine pathway, are in line with a previous study that H_2_S suppresses the expression of IDO1 in murine hepatocellular carcinoma (D. Yang et al., 2019). This consistency across different disease models underscores the fundamental role of H_2_S in modulating KP activity. Furthermore, we also found that the enhancement of necroptosis-mediated neuroinflammation via overexpressing RIPK3 in the hippocampus reversed the correcting role of H_2_S in KP imbalance in the hippocampus of CUMS-exposed rats. Thus, our findings provide compelling evidence that H_2_S attenuates depressive-like behavior of CUMS-exposed rats, which relies on inhibiting the axis of necroptosis-neuroinflammation-KP imbalance.

While our results demonstrate the efficacy of H_2_S in alleviating neuroinflammation and correcting KP imbalance, it is vital to consider its potential advantages compared to other anti-inflammatory strategies. Unlike monoclonal antibodies or NSAIDs that often target a single cytokine or pathway, H_2_S, as an endogenous gasotransmitter, exerts pleiotropic effects across multiple interconnected pathways, including necroptosis, cytokine production, and enzymatic activity within the KP. This multifaceted action may prove more suitable for treating the complex pathophysiology of depression. Furthermore, while general anti-inflammatory drugs may indirectly affect the KP by reducing pro-inflammatory cytokines that drive IDO1 expression, our study provides direct evidence that H_2_S specifically modulates this pathway, potentially through a mechanism that suppresses upstream hippocampal necroptosis, positioning H_2_S as a unique therapeutic candidate capable of simultaneously targeting the core pathological axis of neuroinflammation and metabolic dysregulation in depression.

In summary, the present work demonstrated that CUMS stress triggers hippocampal necroptosis, which leads to the release of pro-inflammatory cytokines, thereby instigating neuroinflammation, and these pro-inflammatory cytokines are known potent inducers of IDO1, shifting the KP towards the neurotoxic branch and causing KP dysregulation. Importantly, H_2_S acts at the apex of this cascade of necroptosis-neuroinflammation-KP imbalance. Our data demonstrate that H_2_S blocks the initiation of necroptosis. This upstream intervention point allows H_2_S to effectively “sever” the entire pathological axis, simultaneously alleviating neuroinflammation and correcting KP imbalance, which is strongly supported by the evidence that the RIPK3 overexpression restored the necroptosis in the presence of H_2_S and reversed all beneficial effects of H_2_S. This finding provides compelling evidence that the regulatory role of H_2_S on inflammation and the KP depend on its necroptosis inhibition. Accordingly, this study provided a solid theoretical basis for establishing H_2_S as a new approach to prevent and treat depression, emphasizing the axis of necroptosis-neuroinflammation-KP imbalance as a promising therapeutic target for the prevention and treatment of depression.

## Data Availability

The original contributions presented in the study are included in the article/Supplementary Material, further inquiries can be directed to the corresponding author.

## Abbreviations

3-HAO, 3-hydroxyanthranilic acid 3,4-dioxygenase; 3-HK, 3-hydroxykynurenine; AAV, Adeno-associated virus; ACMSD, α-amino-β-carboxymuconate-ε-semialdehyde decarboxylase; CUMS, Chronic unpredictable mild stress; i.c.v, Intracerebroventricular; IDO1, Indoleamine 2,3-dioxygenase 1; KYN, Kynurenine; KP, Kynurenine pathway; KMO, Kynurenine 3-monooxygenase; MLKL, Mixed lineage kinase domain-like; RIPK, Receptor-interacting protein kinase; PA, Picolinic acid; QPRT, Quinolinic acid phosphoribosyl transferase; QUIN, Quinolinic acid; TRP, Tryptophan; NAD+, Nicotinamide adenine dinucleotide.

## References

[B1] Aarsland T. I. Leskauskaite I. Midttun Ø. Ulvik A. Ueland P. M. Oltedal L. (2019). The effect of electroconvulsive therapy (ECT) on serum tryptophan metabolites. Brain Stimul. 12 (5), 1135–1142. 10.1016/j.brs.2019.05.018 31176607

[B2] Ali T. Rahman S. U. Hao Q. Li W. Liu Z. Ali Shah F. (2020). Melatonin prevents neuroinflammation and relieves depression by attenuating autophagy impairment through FOXO3a regulation. J. Pineal Res. 69 (2), e12667. 10.1111/jpi.12667 32375205

[B3] Bao P. Gong Y. Wang Y. Xu M. Qian Z. Ni X. (2023). Hydrogen sulfide prevents LPS-induced depression-like behavior through the suppression of NLRP3 inflammasome and pyroptosis and the improvement of mitochondrial function in the hippocampus of mice. Biology 12 (8), 1092. 10.3390/biology12081092 37626978 PMC10451782

[B4] Bauer I. E. Green C. Colpo G. D. Teixeira A. L. Selvaraj S. Durkin K. (2018). A double-blind, randomized, placebo-controlled study of aspirin and N-Acetylcysteine as adjunctive treatments for bipolar depression. J. Clin. Psychiatry 80 (1), 18m12200. 10.4088/JCP.18m12200 30549489

[B5] Brown S. J. Huang X.-F. Newell K. A. (2021). The kynurenine pathway in major depression: what we know and where to next. Neurosci. Biobehav. Rev. 127, 917–927. 10.1016/j.neubiorev.2021.05.018 34029552

[B6] Cao L. Cao X. Zhou Y. Nagpure B. V. Wu Z.-Y. Hu L. F. (2018). Hydrogen sulfide inhibits ATP-induced neuroinflammation and Aβ1-42 synthesis by suppressing the activation of STAT3 and cathepsin S. Brain, Behav. Immun. 73, 603–614. 10.1016/j.bbi.2018.07.005 29981830

[B7] Cao Z.-Y. Liu Y.-Z. Li J.-M. Ruan Y.-M. Yan W.-J. Zhong S.-Y. (2020). Glycyrrhizic acid as an adjunctive treatment for depression through anti-inflammation: a randomized placebo-controlled clinical trial. J. Affect. Disord. 265, 247–254. 10.1016/j.jad.2020.01.048 32090748

[B8] Cervenka I. Agudelo L. Z. Ruas J. L. (2017). Kynurenines: tryptophan's metabolites in exercise, inflammation, and mental health. Sci. (New York, N.Y.) 357 (6349), eaaf9794. 10.1126/science.aaf9794 28751584

[B9] Chen H. Huang X. Zeng C. Sun D. Liu F. Zhang J. (2023). The role of indoleamine 2,3-dioxygenase 1 in early-onset post-stroke depression. Front. Immunol. 14, 1125634. 10.3389/fimmu.2023.1125634 36911716 PMC9998486

[B10] Davis A. K. Barrett F. S. May D. G. Cosimano M. P. Sepeda N. D. Johnson M. W. (2021). Effects of psilocybin-assisted therapy on major depressive disorder: a randomized clinical trial. JAMA Psychiatry 78 (5), 481–489. 10.1001/jamapsychiatry.2020.3285 33146667 PMC7643046

[B11] Deng Y. Zhou M. Wang J. Yao J. Yu J. Liu W. (2021). Involvement of the microbiota-gut-brain axis in chronic restraint stress: disturbances of the kynurenine metabolic pathway in both the gut and brain. Gut Microbes 13 (1), 1–16. 10.1080/19490976.2020.1869501 33535879 PMC7872056

[B12] Fujigaki H. Yamamoto Y. Saito K. (2017). L-Tryptophan-kynurenine pathway enzymes are therapeutic target for neuropsychiatric diseases: focus on cell type differences. Neuropharmacology 112 (Pt B), 264–274. 10.1016/j.neuropharm.2016.01.011 26767951

[B13] Guo B. Zhang M. Hao W. Wang Y. Zhang T. Liu C. (2023). Neuroinflammation mechanisms of neuromodulation therapies for anxiety and depression. Transl. Psychiatry 13 (1), 5. 10.1038/s41398-022-02297-y 36624089 PMC9829236

[B14] Heckmann B. L. Tummers B. Green D. R. (2019). Crashing the computer: apoptosis vs. necroptosis in neuroinflammation. Cell Death Differ. 26 (1), 41–52. 10.1038/s41418-018-0195-3 30341422 PMC6294765

[B15] Hestad K. Alexander J. Rootwelt H. Aaseth J. O. (2022). The role of tryptophan dysmetabolism and quinolinic acid in depressive and neurodegenerative diseases. Biomolecules 12 (7), 998. 10.3390/biom12070998 35883554 PMC9313172

[B16] Hu Y.-B. Zhang Y.-F. Wang H. Ren R.-J. Cui H.-L. Huang W.-Y. (2019). miR-425 deficiency promotes necroptosis and dopaminergic neurodegeneration in Parkinson's disease. Cell Death & Dis. 10 (8), 589. 10.1038/s41419-019-1809-5 31383850 PMC6683159

[B17] Ito Y. Ofengeim D. Najafov A. Das S. Saberi S. Li Y. (2016). RIPK1 mediates axonal degeneration by promoting inflammation and necroptosis in ALS. Sci. (New York, N.Y.) 353 (6299), 603–608. 10.1126/science.aaf6803 27493188 PMC5444917

[B18] Jiang W. Tang Y.-Y. Zhu W.-W. Li C. Zhang P. Li R.-Q. (2021). PI3K/AKT pathway mediates the antidepressant- and anxiolytic-like roles of hydrogen sulfide in streptozotocin-induced diabetic rats *via* promoting hippocampal neurogenesis. Neurotoxicology 85, 201–208. 10.1016/j.neuro.2021.05.016 34087334

[B19] Joisten N. Ruas J. L. Braidy N. Guillemin G. J. Zimmer P. (2021). The kynurenine pathway in chronic diseases: a compensatory mechanism or a driving force? Trends Mol. Med. 27 (10), 946–954. 10.1016/j.molmed.2021.07.006 34373202

[B20] Kang X. Jiang L. Lan F. Tang Y.-Y. Zhang P. Zou W. (2021). Hydrogen sulfide antagonizes sleep deprivation-induced depression- and anxiety-like behaviors by inhibiting neuroinflammation in a hippocampal Sirt1-dependent manner. Brain Res. Bull. 177, 194–202. 10.1016/j.brainresbull.2021.10.002 34624463

[B21] Kim Y.-K. Jeon S. W. (2018). Neuroinflammation and the immune-kynurenine pathway in anxiety disorders. Curr. Neuropharmacol. 16 (5), 574–582. 10.2174/1570159X15666170913110426 28901278 PMC5997870

[B22] Li W. Ali T. He K. Liu Z. Shah F. A. Ren Q. (2021). Ibrutinib alleviates LPS-induced neuroinflammation and synaptic defects in a mouse model of depression. Brain, Behav. Immun. 92, 10–24. 10.1016/j.bbi.2020.11.008 33181270

[B23] Li T.-T. Xin D.-Q. Ke H.-F. Chu X.-L. Zhao Y.-J. Yue S.-W. (2022). L-Cysteine attenuates osteopontin-mediated neuroinflammation following hypoxia-ischemia insult in neonatal mice by inducing S-sulfhydration of Stat3. Acta Pharmacol. Sin. 43 (7), 1658–1669. 10.1038/s41401-021-00794-2 34737419 PMC9253102

[B24] Li X. Yin X. Pang J. Chen Z. Wen J. (2023). Hydrogen sulfide inhibits lipopolysaccharide-based neuroinflammation-induced astrocyte polarization after cerebral ischemia/reperfusion injury. Eur. J. Pharmacol. 949, 175743. 10.1016/j.ejphar.2023.175743 37084816

[B25] Meyer J. H. Cervenka S. Kim M.-J. Kreisl W. C. Henter I. D. Innis R. B. (2020). Neuroinflammation in psychiatric disorders: PET imaging and promising new targets. Lancet. Psychiatry 7 (12), 1064–1074. 10.1016/S2215-0366(20)30255-8 33098761 PMC7893630

[B26] Miller A. H. Maletic V. Raison C. L. (2009). Inflammation and its discontents: the role of cytokines in the pathophysiology of major depression. Biol. Psychiatry 65 (9), 732–741. 10.1016/j.biopsych.2008.11.029 19150053 PMC2680424

[B27] Mithaiwala M. N. Santana-Coelho D. Porter G. A. O'Connor J. C. (2021). Neuroinflammation and the kynurenine pathway in CNS disease: molecular mechanisms and therapeutic implications. Cells 10 (6), 1548. 10.3390/cells10061548 34205235 PMC8235708

[B28] Ou W. Chen Y. Ju Y. Ma M. Qin Y. Bi Y. (2023). The kynurenine pathway in major depressive disorder under different disease states: a systematic review and meta-analysis. J. Affect. Disord. 339, 624–632. 10.1016/j.jad.2023.07.078 37467793

[B29] Paul E. R. Schwieler L. Erhardt S. Boda S. Trepci A. Kämpe R. (2022). Peripheral and central kynurenine pathway abnormalities in major depression. Brain, Behav. Immun. 101, 136–145. 10.1016/j.bbi.2022.01.002 34999196 PMC9045681

[B30] Picon C. Jayaraman A. James R. Beck C. Gallego P. Witte M. E. (2021). Neuron-specific activation of necroptosis signaling in multiple sclerosis cortical grey matter. Acta Neuropathol. 141 (4), 585–604. 10.1007/s00401-021-02274-7 33569629 PMC7952371

[B31] Pinna A. Kistowska J. Pałasz A. (2025). An Enigma of brain gasotransmitters: hydrogen sulfide and depression. Neuromolecular Med. 27 (1), 58. 10.1007/s12017-025-08880-y 40779086 PMC12334376

[B32] Réus G. Z. Manosso L. M. Quevedo J. Carvalho A. F. (2023). Major depressive disorder as a neuro-immune disorder: origin, mechanisms, and therapeutic opportunities. Neurosci. Biobehav. Rev. 155, 105425. 10.1016/j.neubiorev.2023.105425 37852343

[B33] Schwieler L. Larsson M. K. Skogh E. Kegel M. E. Orhan F. Abdelmoaty S. (2015). Increased levels of IL-6 in the cerebrospinal fluid of patients with chronic schizophrenia--significance for activation of the kynurenine pathway. J. Psychiatry & Neurosci. JPN 40 (2), 126–133. 10.1503/jpn.140126 25455350 PMC4354818

[B34] Schwieler L. Samuelsson M. Frye M. A. Bhat M. Schuppe-Koistinen I. Jungholm O. (2016). Electroconvulsive therapy suppresses the neurotoxic branch of the kynurenine pathway in treatment-resistant depressed patients. J. Neuroinflammation 13 (1), 51. 10.1186/s12974-016-0517-7 26925576 PMC4772340

[B35] Setiawan E. Wilson A. A. Mizrahi R. Rusjan P. M. Miler L. Rajkowska G. (2015). Role of translocator protein density, a marker of neuroinflammation, in the brain during major depressive episodes. JAMA Psychiatry 72 (3), 268–275. 10.1001/jamapsychiatry.2014.2427 25629589 PMC4836849

[B36] Sun Y. Chen X. Ou Z. Wang Y. Chen W. Zhao T. (2022). Dysmyelination by oligodendrocyte-specific ablation of Ninj2 contributes to depressive-like behaviors. Adv. Sci. 9 (3), e2103065. 10.1002/advs.202103065 34787377 PMC8787401

[B37] Tang X. Wu J. Ding C.-K. Lu M. Keenan M. M. Lin C.-C. (2016). Cystine deprivation triggers programmed necrosis in VHL-deficient renal cell carcinomas. Cancer Res. 76 (7), 1892–1903. 10.1158/0008-5472.CAN-15-2328 26833124 PMC4873412

[B38] Thadathil N. Nicklas E. H. Mohammed S. Lewis T. L. Richardson A. Deepa S. S. (2021). Necroptosis increases with age in the brain and contributes to age-related neuroinflammation. GeroScience 43 (5), 2345–2361. 10.1007/s11357-021-00448-5 34515928 PMC8599532

[B39] Tolkien K. Bradburn S. Murgatroyd C. (2019). An anti-inflammatory diet as a potential intervention for depressive disorders: a systematic review and meta-analysis. Clin. Nutr. Edinb. Scotl. 38 (5), 2045–2052. 10.1016/j.clnu.2018.11.007 30502975

[B40] Wang Y. Wang S. Xin Y. Zhang J. Wang S. Yang Z. (2021). Hydrogen sulfide alleviates the anxiety-like and depressive-like behaviors of type 1 diabetic mice *via* inhibiting inflammation and ferroptosis. Life Sci. 278, 119551. 10.1016/j.lfs.2021.119551 33945828

[B41] Xue C. Li G. Zheng Q. Gu X. Shi Q. Su Y. (2023). Tryptophan metabolism in health and disease. Cell Metab. 35 (8), 1304–1326. 10.1016/j.cmet.2023.06.004 37352864

[B42] Yan E. B. Frugier T. Lim C. K. Heng B. Sundaram G. Tan M. (2015). Activation of the kynurenine pathway and increased production of the excitotoxin quinolinic acid following traumatic brain injury in humans. J. Neuroinflammation 12, 110. 10.1186/s12974-015-0328-2 26025142 PMC4457980

[B43] Yang D. Li T. Li Y. Zhang S. Li W. Liang H. (2019). H2S suppresses indoleamine 2, 3-dioxygenase 1 and exhibits immunotherapeutic efficacy in murine hepatocellular carcinoma. J. Exp. & Clin. Cancer Res. CR 38 (1), 88. 10.1186/s13046-019-1083-5 30777103 PMC6380069

[B44] Yang S.-Q. Tang Y.-Y. Zeng D. Tian Q. Wei H.-J. Wang C.-Y. (2022). Sodium hydrosulfide reverses β2-microglobulin-induced depressive-like behaviors of male Sprague-Dawley rats: involving improvement of synaptic plasticity and enhancement of Warburg effect in hippocampus. Behav. Brain Res. 417, 113562. 10.1016/j.bbr.2021.113562 34499939

[B45] Yao D. Li R. Hao J. Huang H. Wang X. Ran L. (2023). Melatonin alleviates depression-like behaviors and cognitive dysfunction in mice by regulating the circadian rhythm of AQP4 polarization. Transl. Psychiatry 13 (1), 310. 10.1038/s41398-023-02614-z 37802998 PMC10558463

[B46] Yao H. Zhang D. Yu H. Yuan H. Shen H. Lan X. (2023). Gut microbiota regulates chronic ethanol exposure-induced depressive-like behavior through hippocampal NLRP3-mediated neuroinflammation. Mol. Psychiatry 28 (2), 919–930. 10.1038/s41380-022-01841-y 36280756 PMC9908543

[B47] Yu Z. Jiang N. Su W. Zhuo Y. (2021). Necroptosis: a novel pathway in neuroinflammation. Front. Pharmacol. 12, 701564. 10.3389/fphar.2021.701564 34322024 PMC8311004

[B48] Yuan J. Amin P. Ofengeim D. (2019). Necroptosis and RIPK1-mediated neuroinflammation in CNS diseases. Nat. Rev. Neurosci. 20 (1), 19–33. 10.1038/s41583-018-0093-1 30467385 PMC6342007

[B49] Zhang F. Zhu X. Yu P. Sheng T. Wang Y. Ye Y. (2022). Crocin ameliorates depressive-like behaviors induced by chronic restraint stress *via* the NAMPT-NAD+-SIRT1 pathway in mice. Neurochem. Int. 157, 105343. 10.1016/j.neuint.2022.105343 35490894

[B50] Zhao H. Pan P. Yang Y. Ge H. Chen W. Qu J. (2017). Endogenous hydrogen sulphide attenuates NLRP3 inflammasome-mediated neuroinflammation by suppressing the P2X7 receptor after intracerebral haemorrhage in rats. J. Neuroinflammation 14 (1), 163. 10.1186/s12974-017-0940-4 28821266 PMC5563049

[B51] Zhao Y. Li T. Jiang Z. Gai C. Yu S. Xin D. (2024). The miR-9-5p/CXCL11 pathway is a key target of hydrogen sulfide-mediated inhibition of neuroinflammation in hypoxic ischemic brain injury. Neural Regen. Res. 19 (5), 1084–1094. 10.4103/1673-5374.382860 37862212 PMC10749591

[B52] Zunszain P. A. Anacker C. Cattaneo A. Choudhury S. Musaelyan K. Myint A. M. (2012). Interleukin-1β: a new regulator of the kynurenine pathway affecting human hippocampal neurogenesis. Neuropsychopharmacol. Official Publ. Am. Coll. Neuropsychopharmacol. 37 (4), 939–949. 10.1038/npp.2011.277 22071871 PMC3280640

