# Supplementary material for: Hydrogen sulphide alleviates depression-like behaviors by suppressing the hippocampal necroptosis-neuroinflammation-KP imbalance axis
Source: Front Pharmacol. 2025 Oct 24;16:1691204. doi: 10.3389/fphar.2025.1691204 (PMC12592148; doi:10.3389/fphar.2025.1691204)
Supplement: Supplementary file 1 [file Supplementaryfile1.docx]

**Supplementary Fig. 1**


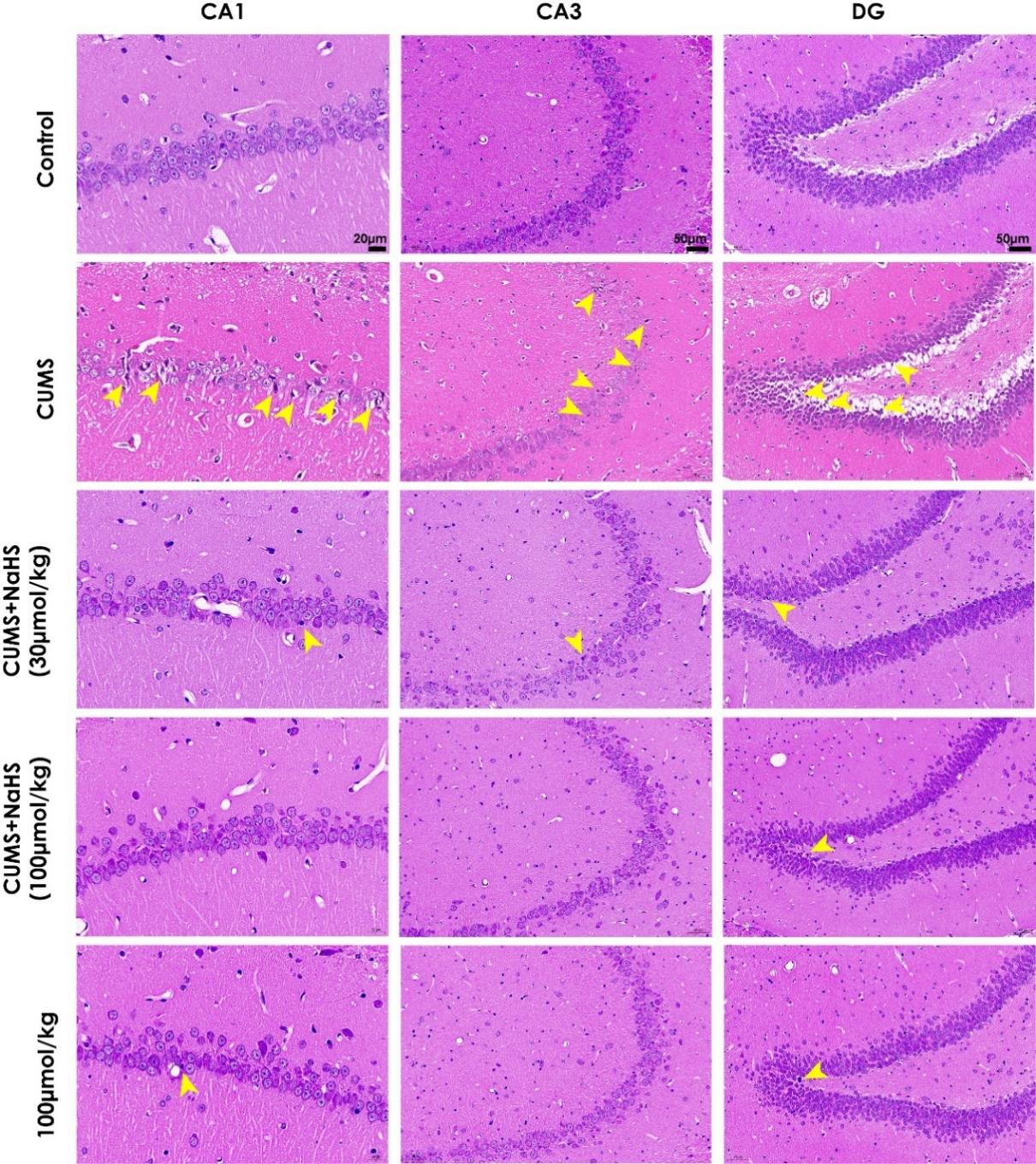


**Supplementary Figure 1. Effects of NaHS** **on the histopathological changes in the hippocampus of CUMS-exposed rats.** Rats were exposed to CUMS for 4 weeks and treated with NaHS (30, 100 μmol/kg, i.p.) for 2 weeks. The histopathological changes in the hippocampus (CA1, CA3, DG region) of CUMS-exposed rats was detected by HE staining (scale bar = 20 or 50 μm). The yellow arrow indicates the necrotic cells.

**Supplementary Fig. 2**


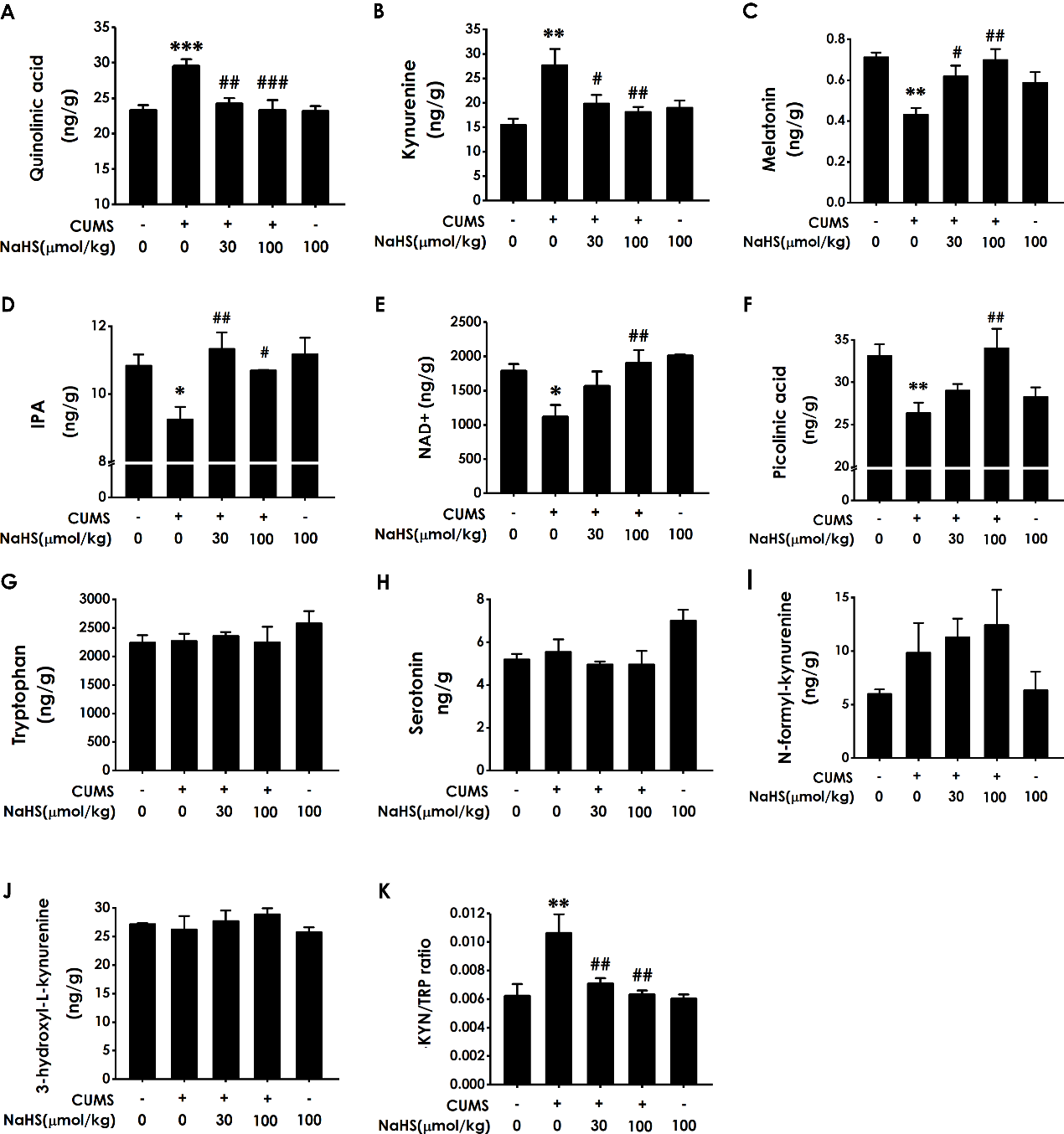


**Supplementary Figure 2. Effect of NaHS on the disorder of kynurenine metabolism in the hippocampus of CUMS-exposed rats.** The concentration of quinolinic acid (A), kynurenine (B), melatonin (C), IPA (D), NAD+ (E), picolinic acid (F), tryptophan (G), serotonin (H), N-formyl-kynurenine (I), 3-hydroxy-kynurenine (J), and KYN/TRP ratio (K) in the hippocampus of rats were measured by LC-MS/MS. The KYN/TRP ratio (K) represents IDO1 activity. Values are expressed as mean ± SEM (𝑛=6-7/group). ^*^𝑃 < 0.05, ^**^𝑃 < 0.01, ^***^𝑃 < 0.001, versus the control group; ^#^𝑃 < 0.05, ^##^𝑃 < 0.01, ^###^𝑃 < 0.001, versus the CUMS-treated group.

**Supplementary Fig. 3**


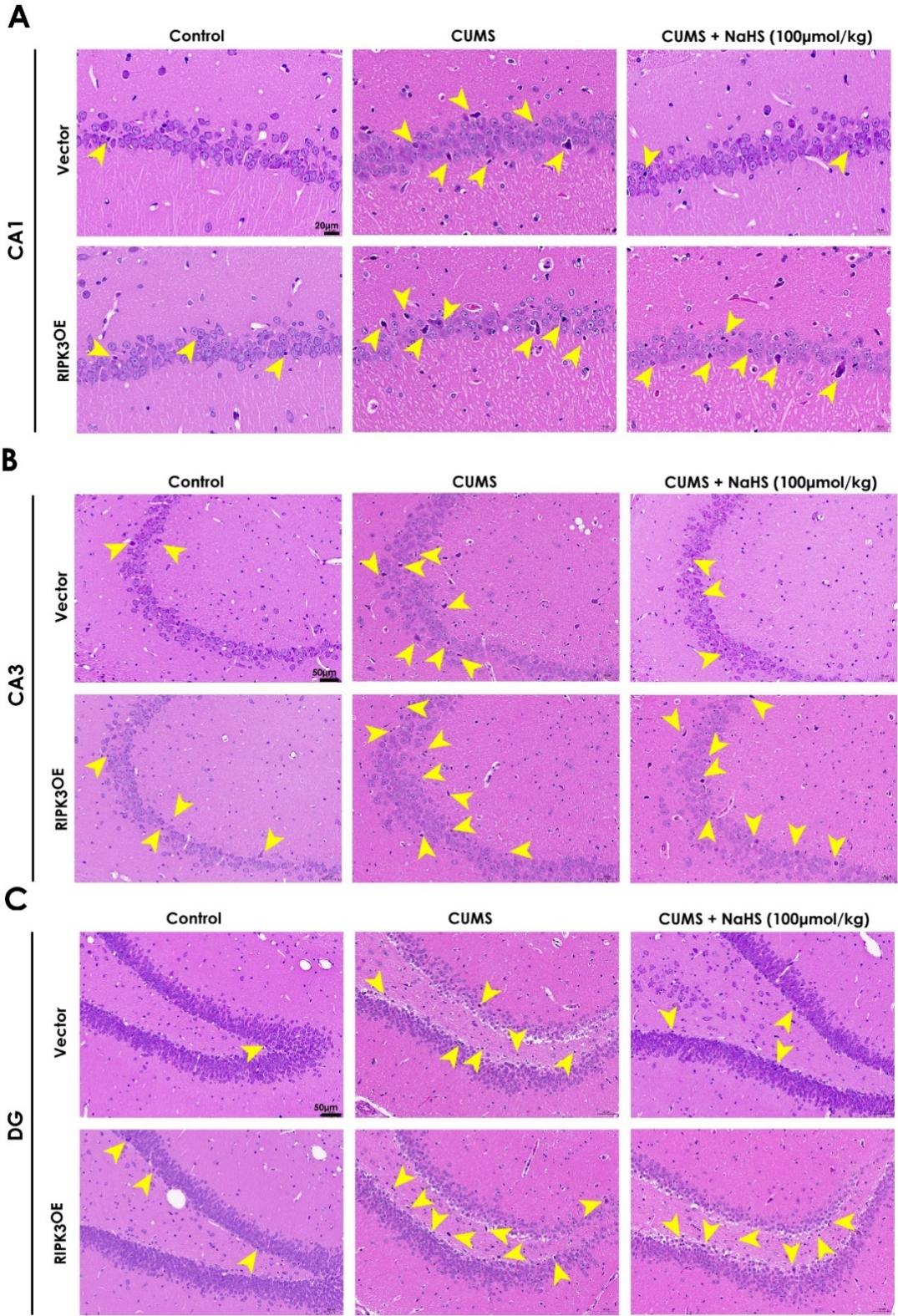


**Supplementary Figure. 3** **Effect of AAV-RIPK3 on NaHS-alleviated the pathological damage in the hippocampus of CUMS-exposed rats.** Rats were injected with vector or AAV-RIPK3 into the hippocampus for once. One week after the injection of AAV-RIPK3, rats were exposed to CUMS for 4 weeks and treated with NaHS (100 μmol/kg, i.p.) for 2 weeks. The histopathological changes in the hippocampus (CA1, CA3, DG region) of rats was detected by HE staining (scale bar = 20 or 50 μm). The yellow arrow indicates the necrotic cells.

**Supplementary Fig. 4**


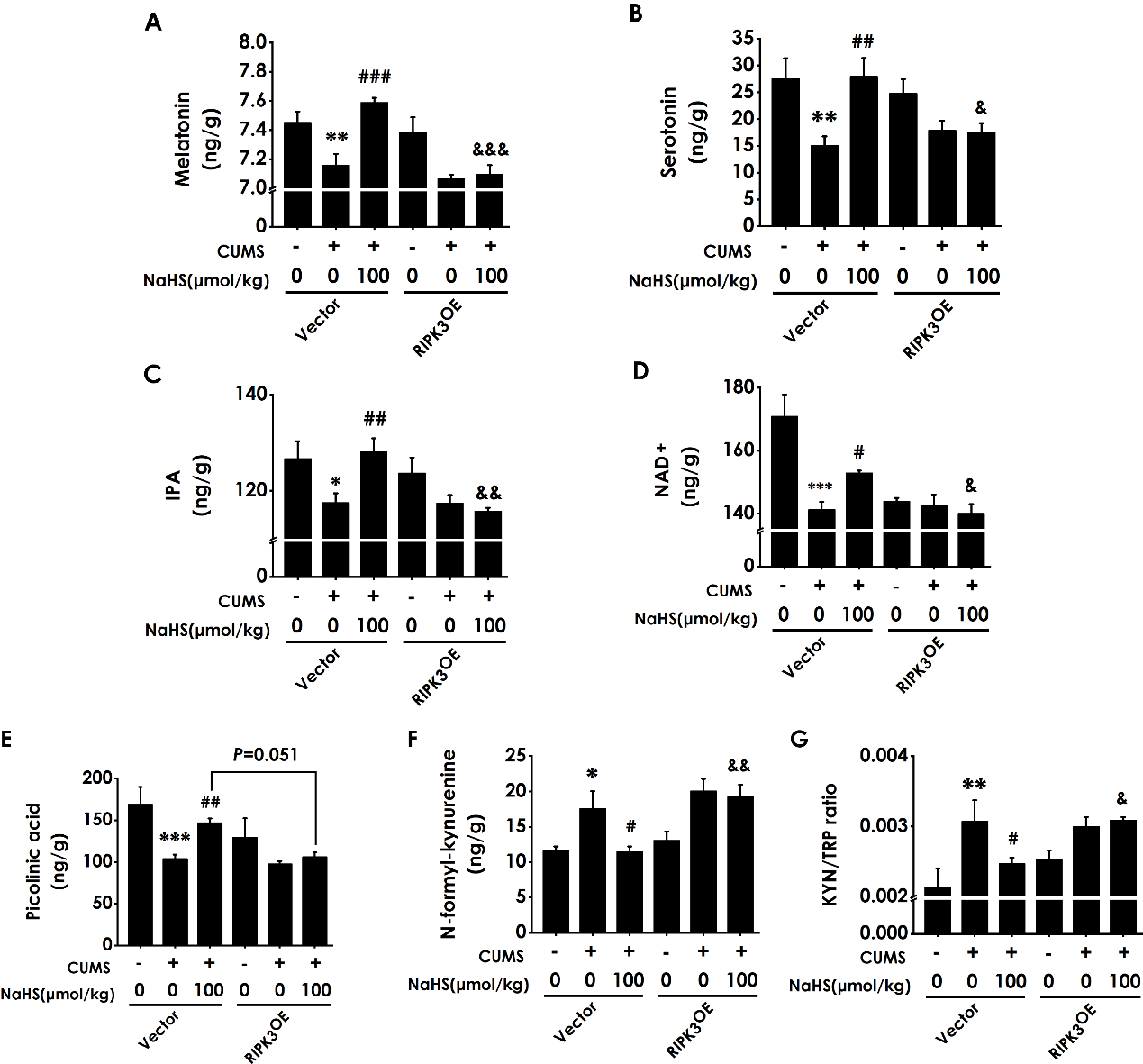


**Supplementary Figure 4. Effect of AAV-RIPK3 on NaHS-corrected the levels of kynurenine metabolites in the hippocampus of CUMS-exposed rats.** Rats were injected with vector or AAV-RIPK3 into the hippocampus for once. One week after the injection of AAV-RIPK3, rats were exposed to CUMS for 4 weeks and treated with NaHS (100 μmol/kg, i.p.) for 2 weeks. The concentration of melatonin (A), serotonin (B), IPA (C), NAD+ (D), picolinic acid (E), N-formyl-kynurenine (F), and KYN/TRP (G) ratio in the hippocampus of rats were measured by LC-MS/MS. The KYN/TRP (G) ratio represents IDO1 activity. Values are expressed as mean ± SEM (𝑛=3/group). ^*^𝑃 < 0.05, ^*^𝑃 < 0.01, ^***^𝑃 < 0.001 versus the vector group; ^#^𝑃 < 0.05, ^##^𝑃 < 0.01, ^###^𝑃 < 0.001, versus co-treated with CUMS and vector group; ^&&^𝑃 < 0.05, ^&&^𝑃 < 0.01, ^&&&^𝑃 < 0.001, versus co-treated with CUMS, NaHS, and vector group.
